# The GATOR2 Component Wdr24 Regulates TORC1 Activity and Lysosome Function

**DOI:** 10.1371/journal.pgen.1006036

**Published:** 2016-05-11

**Authors:** Weili Cai, Youheng Wei, Michal Jarnik, John Reich, Mary A. Lilly

**Affiliations:** Cell Biology and Neurobiology Branch, National Institute of Child Health and Human Development, National Institutes of Health, Bethesda, Maryland, United States of America; University of Texas Southwestern, UNITED STATES

## Abstract

TORC1 is a master regulator of metabolism in eukaryotes that responds to multiple upstream signaling pathways. The GATOR complex is a newly defined upstream regulator of TORC1 that contains two sub-complexes, GATOR1, which inhibits TORC1 activity in response to amino acid starvation and GATOR2, which opposes the activity of GATOR1. While the GATOR1 complex has been implicated in a wide array of human pathologies including cancer and hereditary forms of epilepsy, the *in vivo* relevance of the GATOR2 complex remains poorly understood in metazoans. Here we define the *in vivo* role of the GATOR2 component Wdr24 in *Drosophila*. Using a combination of genetic, biochemical, and cell biological techniques we demonstrate that Wdr24 has both TORC1 dependent and independent functions in the regulation of cellular metabolism. Through the characterization of a null allele, we show that Wdr24 is a critical effector of the GATOR2 complex that promotes the robust activation of TORC1 and cellular growth in a broad array of *Drosophila* tissues. Additionally, epistasis analysis between *wdr24* and genes that encode components of the GATOR1 complex revealed that Wdr24 has a second critical function, the TORC1 independent regulation of lysosome dynamics and autophagic flux. Notably, we find that two additional members of the GATOR2 complex, Mio and Seh1, also have a TORC1 independent role in the regulation of lysosome function. These findings represent a surprising and previously unrecognized function of GATOR2 complex components in the regulation of lysosomes. Consistent with our findings in *Drosophila*, through the characterization of a *wdr24*^*-/-*^ knockout HeLa cell line we determined that Wdr24 promotes lysosome acidification and autophagic flux in mammalian cells. Taken together our data support the model that Wdr24 is a key effector of the GATOR2 complex, required for both TORC1 activation and the TORC1 independent regulation of lysosomes.

## Author Summary

TORC1 is a conserved multi-protein complex that regulates metabolism and cell growth in response to many upstream inputs including nutrient availability. When amino acids are limiting, the GATOR1 complex inhibits TORC1 activation. The inhibition of TORC1 slows cellular metabolism and promotes cell survival during times of protein scarcity. A second critical response to amino acid limitation is the activation of autophagy. During autophagy cells degrade intracellular components in specialized membrane-bound organelles called autolysosomes that are formed when lysosomes fuse with autophagosomes. In times of nutrient stress, the process of autophagy allows proteins and other building blocks of the cell to be broken down and repurposed for vital cellular functions. Here we demonstrate that Wdr24, a component of the multi-protein GATOR2 complex, has a dual role in the regulation of cellular metabolism in *Drosophila*. First, Wdr24 is required to oppose the activity of the GATOR1 complex, thus activating TORC1 in a broad array of *Drosophila* tissues. Second, Wdr24 promotes the acidification of lysosomes and thus facilitates autophagic flux. Our data support the model that Wdr24 uses both TORC1 dependent and independent pathways to regulate cellular metabolism.

## Introduction

In metazoans multiple conserved signaling pathways control the integration of metabolic and developmental processes. TORC1 is an evolutionarily conserved multi-protein complex that regulates metabolism and cell growth in response to an array of upstream inputs including nutrient availability, growth factors and intracellular energy levels [1]. The catalytic component of TORC1 is the serine/threonine kinase Target of Rapamycin (TOR). When nutrients are abundant, TORC1 activity promotes translation, ribosome biogenesis as well as other pathways associated with anabolic metabolism and cell growth. However, when nutrients or other upstream activators are limiting, TORC1 activity is inhibited triggering catabolic metabolism and autophagy [2].

The Seh1 associated/GTPase-activating protein toward Rags (SEA/GATOR) complex is a newly identified upstream regulator of TORC1 that can be divided into two putative sub-complexes GATOR1 and GATOR2 [3–5]. The GATOR1 complex, known as the Iml1 complex or the Seh1 Associated Complex Inhibits TORC1 (SEACIT) in yeast, inhibits TORC1 activity in response to amino acid limitation [3,5,6]. SEACIT/GATOR1 contains three proteins Npr2/Nprl2, Npr3/Nprl3 and Iml1/DEPDC5. Recent evidence, from yeast and mammals, indicates that the components of the SEACIT/GATOR1 complex function through the Rag GTPases to inhibit TORC1 activity [3,5]. Notably, Nprl2 and DEPDC5 are tumor suppressor genes while mutations in DEPDC5 are a leading cause of hereditary focal epilepsies [7–16].

The GATOR2 complex, which is referred to as Seh1 Associated Complex Activates TORC1 (SEACAT) in yeast, activates TORC1 by opposing the activity of GATOR1 [3,5,17,18]. The SEACAT/GATOR2 complex is comprised of five proteins, Seh1, Sec13, Sea4/Mio, Sea2/WDR24, and Sea3/WDR59. Computational analysis indicates that multiple components of the GATOR2 complex have structural features characteristic of coatomer proteins and membrane tethering complexes [4,19]. In line with the structural similarity to proteins that influence membrane dynamics, in *Drosophila* the GATOR2 subunits Mio and Seh1 localize to multiple endomembrane compartments including lysosomes, the site of TORC1 regulation, and autolysosomes [18]. In metazoans, members of the Sestrin and Castor family of proteins bind to and inhibit the GATOR2 complex in response to leucine and arginine starvation respectively [20–25]. This interaction is proposed to inhibit TORC1 activity through the derepression of the GATOR1 complex [22,23,26]. However, how GATOR2 opposes GATOR1 activity, thus allowing for the robust activation of TORC1, remains unknown. Additionally, the role of the GATOR2 complex in the regulation of both the development and physiology of multicellular animals remains poorly defined.

Recent evidence from *Drosophila* indicates that the requirement for the GATOR2 complex may be context specific in multicellular animals [18]. In *Drosophila*, null alleles of the GATOR2 components *mio* and *seh1* are viable but female sterile [27,28]. Surprisingly, somatic tissues from *mio* and *seh1* mutants exhibit little if any reductions in cell size and have nearly normal levels of TORC1 activity [18]. In contrast, TORC1 activity is dramatically decreased in ovaries from *mio* and *seh1* mutant females [18]. This decrease in TORC1 activity is accompanied by the activation of catabolic metabolism in the female germ line, a dramatic reduction in egg chamber growth and difficulties maintaining the meiotic cycle [27,28]. Thus, there is a surprising tissue specific requirement for the GATOR2 components Mio and Seh1 during oogenesis. However, the *in vivo* role of the other members of the GATOR2 complex in the regulation of cellular metabolism remains undefined.

Here we define the *in vivo* requirement for the GATOR2 component Wdr24 in *Drosophila*. We find that Wdr24 has two distinct functions. First, Wdr24 is a critical effector of the GATOR2 complex that promotes TORC1 activity and cellular growth in a broad array of tissues. Second, Wdr24 is required for the TORC1 independent regulation of lysosome function and autophagic flux. Notably, two additional members of the GATOR2 complex, Mio and Seh1, also have a TORC1 independent role in the regulation of lysosome function. Taken together our data support the model that multiple components of the GATOR2 complex have both TORC1 dependent and independent roles in the regulation of cellular metabolism.

## Results

### The GATOR2 component Wdr24 localizes to lysosomes and autolysosomes

**Fig 1:**
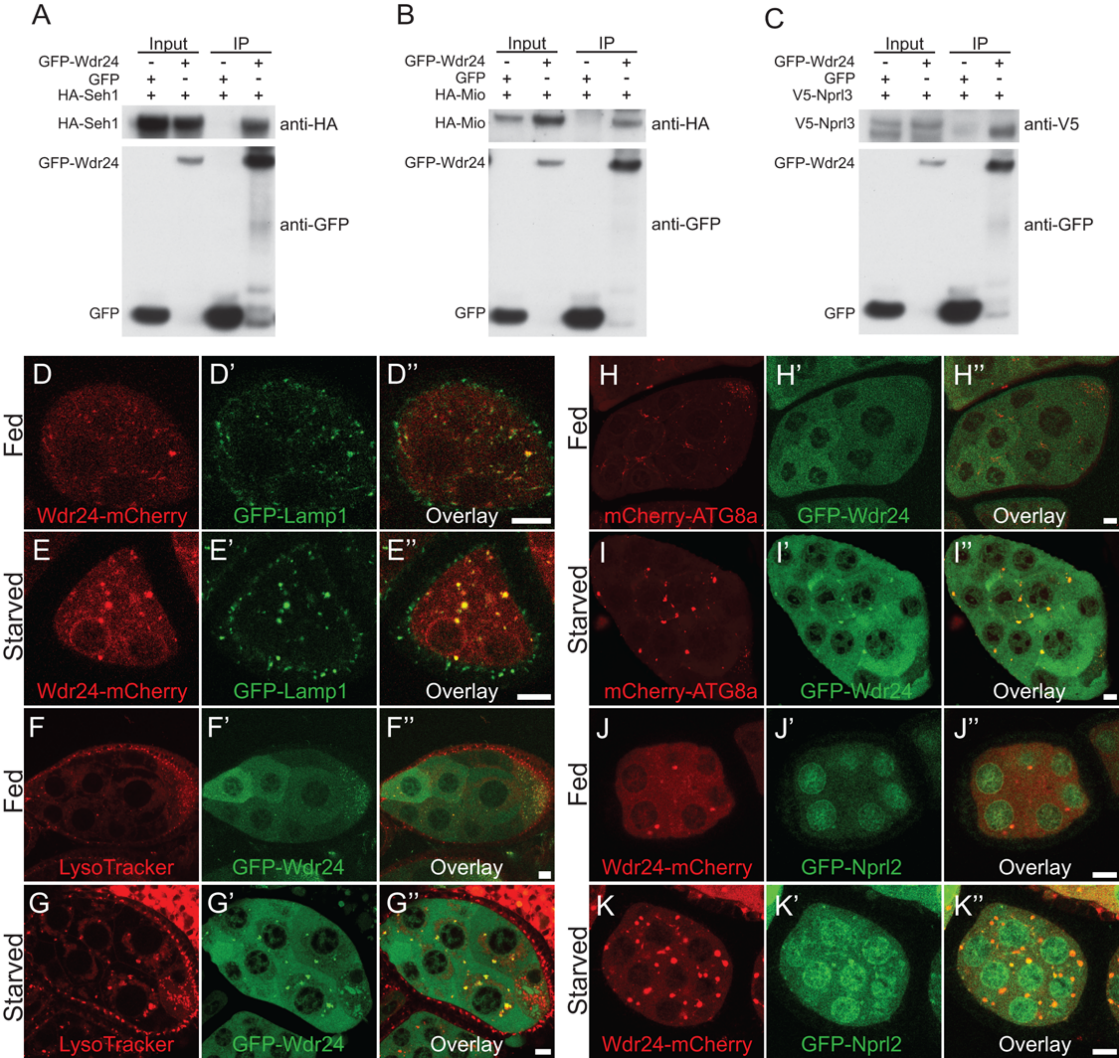
Wdr24 associates with GATOR complex components and localizes to lysosomes and autolysosomes. (A-C) S2 cells were co-transfected with HA-tagged Seh1, HA-tagged Mio, V5-tagged Nprl3 and GFP-tagged Wdr24 or GFP (control) plasmids. Cells were lysed and immunoprecipitated with GFP antibody. Cell lysates (input) and immunoprecipitates (IP) were detected by Western blot using HA, V5 and GFP antibodies. (D-K) Live cell imaging of *Drosophila* egg chambers from females cultured on standard fly medium (fed) or on 20% sucrose (starved). (D-E”) Wdr24-mCherry co-localizes with GFP-Lamp1. (F-G”) GFP-Wdr24 co-localizes with LysoTracker. (H-I”) GFP-Wdr24 co-localizes with mCherry-Atg8 under starvation conditions. (J-K”) Wdr24-mCherry co-localizes with GFP-Nprl2. Size bar is 10 μm.

Sea2/Wdr24 is a conserved component of the SEA/GATOR complex in yeast and mammals and has been implicated in the regulation of TORC1 activity and autophagy [3–5]. The genome of *Drosophila melanogaster* contains a single Sea2/Wdr24 homolog encoded by the gene CG7609 that shares 25% identity and 44% similarity to yeast Sea2 and 37% identity and 54% similarity to the human homolog WDR24. In the work presented here *Drosophila* CG7609 is referred to as Wdr24. To confirm the association of Wdr24 (CG7609) with other components of the SEA/GATOR complex in *Drosophila*, we co-expressed GFP-Wdr24 with HA-Mio, HA-Seh1, and V5-Nprl3 in S2 cells and found that GFP-Wdr24 co-immunoprecipitated with all three SEA/GATOR complex components (Fig 1A–1C). Moreover, we found that the immunoprecipitation of a FLAG-Mio-HA tagged protein expressed in the female germ line co-immunoprecipitated all 7 additional members of the GATOR complex strongly suggesting that the association of these proteins is conserved in *Drosophila* (S1 Table). Thus, as is observed in mammals and yeast, Wdr24 is a conserved component of the SEA/GATOR complex in *Drosophila*. We recognize, however, that these data do not rule out the possibility Wdr24 is present in additional complexes.

Next, we wanted to determine the intracellular localization of Wdr24. Notably, the GATOR2 components Mio and Seh1 localize to lysosomes, the site of TORC1 activation, and autolysosomes [3,18]. Consistent with the localization of Mio and Seh1 we found Wdr24-mCherry expressed in the female germ line co-localized with GFP-Lamp1, a marker for both lysosomes and autolysosomes, under fed and starved conditions (Fig 1D–1E”). Additionally, we examined if Wdr24 co-localized with LysoTracker, a dye that marks acidic compartments including late endosomes, lysosomes and autolysosome [29]. Similar to GFP-Lamp1, GFP-Wdr24 co-localized with LysoTracker (Fig 1F–1G”). We noted in both sets of experiments that there was a substantial increase in the Wdr24-mCherry / GFP-Lamp1 and Wdr24/LysoTracker positive puncta in ovaries from starved versus fed females. We reasoned that the increased number and size of the puncta under starvation conditions was likely the result of the onset of autophagy and the production of autolysosomes. Consistent with this idea, GFP-Wdr24 co-localized with mCherry-Atg8a, a component of autophagosomes and autolysosomes, under starvation conditions. However, this co-localization was not observed when ovaries were taken from females cultured in nutrient rich conditions (Fig 1H–1I”). Finally, Wdr24-mCherry colocalizes to puncta with the GATOR1 component GFP-Nprl2 when co-expresses in the female germline under conditions of amino acid starvation. Minimal co-localization was observed under nutrient replete conditions (Fig 1J–1K”). Taken together these data demonstrate that as is observed with the GATOR2 components Mio and Seh1, the Wdr24 protein localizes to lysosomes, the site of TORC1 regulation, and to autolysosomes.

### Wdr24 promotes TORC1 activity and growth in germline and somatic cells

To examine the *in vivo* function of Wdr24, we obtained the *wdr24* mutant *CG7609*^*1*^ from the Bloomington Stock Center, which contains a 1.3 kb deletion removing 1163 bp of coding region including the start codon (S1A Fig). In this study, the *CG7609*^*1*^ mutant is referred to as *wdr24*^*1*^. We find that *wdr24* is not required for viability in *Drosophila*. RT-PCR demonstrated that the *wdr24* mRNA is present in WT (wild type) but not in *wdr24*^*1*^ homozygous or *wdr24*^*1*^*/Df* adults (S1B Fig). These data confirm that *wdr24*^*1*^ is a null allele.

**Fig 2:**
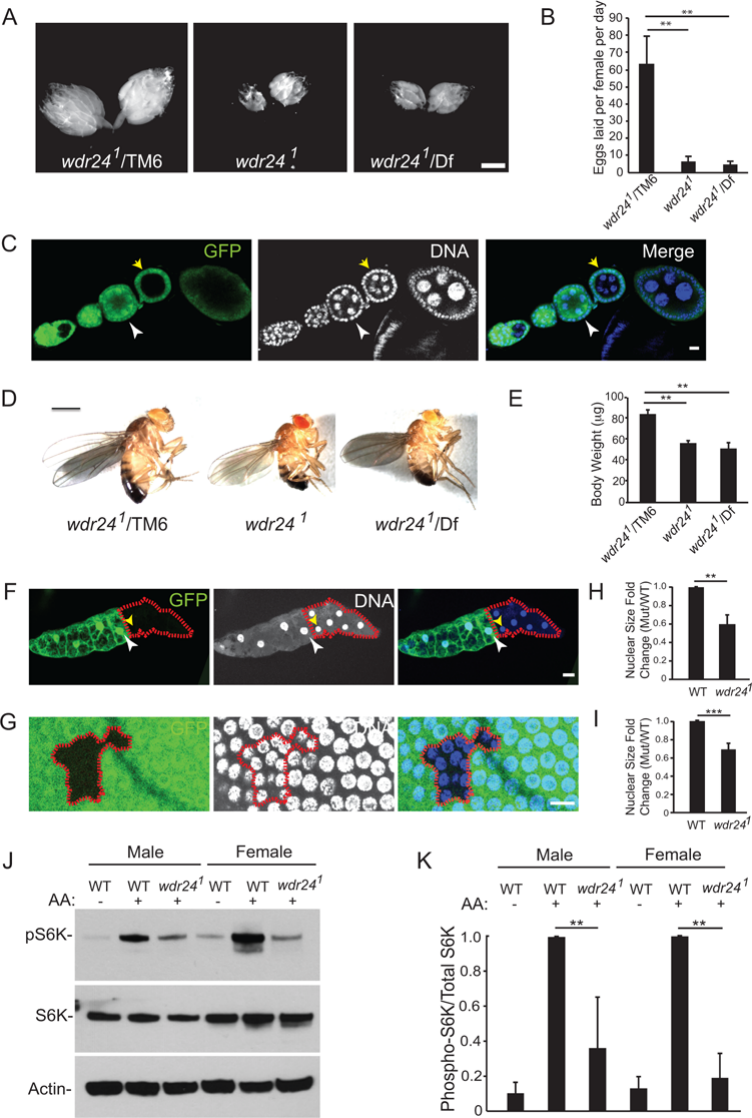
Wdr24 promotes TORC1 activity and cell growth in both germline and somatic cells. (A) Dissected ovaries from control *wdr24*^*1*^/*TM6* (control), *wdr24*^*1*^ and *wdr24*^*1*^*/Df* females. Size bar is 100 μm. (B) Bar graph shows the number of eggs laid by control *wdr24*^*1*^/*TM6*, *wdr24*^*1*^ and *wdr24*^*1*^*/Df* females. Error bars represent the standard deviation for three independent experiments. ** p value < 0.01 (C) Ovariole containing a *wdr24*^*1*^ mutant germline clone stained with anti-GFP and DAPI. Egg chambers containing *wdr24*^*1*^ germline clones are marked by the absence of GFP. Note that the *wdr24*^*1*^ mutant egg chamber (yellow arrow) is smaller than a younger WT egg chamber (white arrowhead). Size bar is 10 μm (D) Representative images of control *wdr24*^*1*^/*TM6*, *wdr24*^*1*^ and *wdr24*^*1*^*/Df* adult males. Size bar is 100 μm. (E) Quantification of body weights of *wdr24*^*1*^/*TM6*, *wdr24*^*1*^ and *wdr24*^*1*^*/Df* adult males. Error bars represent the standard deviation for three independent experiments (8 males per group). **p value < 0.01 (F and G) Somatically derived cells from an adult fat body (F) and follicle cells (G) from a stage 10B egg chamber stained with anti-GFP and DAPI. The *wdr24*^*1*^ mutant cells are marked by the absence of GFP and are outlined by a red line. Note that *wdr24*^*1*^ mutant cells have a smaller nuclear size suggesting decreased ploidy. Size bar is 10 μm (H and I) Quantification of nuclear size fold change of *wdr24*^*1*^ mutant cells compared to wild type cells from adult fat bodies (H) and follicle cells (I). Error bars represent the standard deviation from 4 individual fat body clones and 9 individual follicle cell clones. **p value < 0.01, *** p value < 0.001 (J) Proteins isolated from WT, *wdr24*^*1*^ and starved WT (positive control) females and males were analyzed by Western blot probed with pS6K and S6K antibodies (K) Quantification of phospho-S6K levels relative to the total S6K. Error bars represent the standard deviation for three independent experiments. **p value < 0.01.

In *mio* and *seh1* mutants, the constitutive inhibition of TORC1 in the female germ line results in female sterility and a dramatic reduction in egg chamber growth [18]. Similarly, *wdr24*^*1*^ females have small ovaries and exhibit a 90% reduction in eggs laid per day relative to heterozygous controls (Fig 2A and 2B, S1C and S1D Fig). Homozygous germline clones of *wdr24*^*1*^ result in reduced egg chamber growth, indicating that *wdr24* acts cell autonomously in the germ line to promote growth (Fig 2C). Surprisingly, however, we did not observe an oocyte loss phenotype in *wdr24*^*1*^ mutant egg chambers, as has been reported in a high percentage of the egg chambers from both *mio* and *seh1* mutant females (S2 Fig) [27,28]. Finally, the small ovary and egg laying deficits of *wdr24*^*1*^ females were rescued by expressing GFP-Wdr24 in the *wdr24*^*1*^ mutant background using the germline specific driver Nanos-Gal4 (S1C and S1D Fig). These data confirm that Wdr24 is required for ovary growth and female fertility. Thus, while the GATOR2 components Mio, Seh1 and Wdr24 are required for female fertility and egg chamber growth, our data suggest that individual GATOR2 subunits have unique functions, and/or make differential contributions to the regulation of oocyte development.

Next we examined multiple somatic tissues to determine if there is a requirement for Wdr24 outside of the female germ line. Under standard culture conditions, null mutations of *mio* and *seh1* do not result in dramatic changes in cell size or TORC1 activity in somatic tissues [18]. In contrast, we noted that *wdr24*^*1*^ mutant adults appeared smaller than their heterozygous siblings (Fig 2D). Consistent with this observation, well-fed *wdr24*^*1*^ and *wdr24*^*1*^/*Df* mutant males weigh approximately 30% less than sibling controls (Fig 2E). The decreased body weight phenotype was rescued by expressing GFP-tagged Wdr24 in the *wdr24*^*1*^ mutant background using the ubiquitous driver, Ubi-Gal4 (S3 Fig). Thus, *wdr24* mutant adults have an overall decrease in body size and weight. In order to determine if the effects of *wdr24* on cell growth were cell autonomous, we generated homozygous mutant clones of the *wdr24*^*1*^ null allele. We found that *wdr24*^*1*^ homozygous mutant clones generated in both the adult fat body and the somatically derived follicle cells have a decreased nuclear size relative to adjacent wild-type heterozygous cells (Fig 2F–2I). From these results we infer that there is a cell autonomous requirement for *wdr24* to promote cellular growth in multiple somatic tissues. In summary, we find that there is a critical requirement for the GATOR2 component Wdr24 in promoting cell growth in both germline and somatic tissues of *Drosophila*.

In order to determine if the decreased growth observed in *wdr24*^*1*^ mutants is accompanied by decreased TORC1 activity, we examined the phosphorylation status of S6 kinase, a downstream TORC1 target [30] in wild-type and *wdr24* mutant males and females. We found that *wdr24*^*1*^ mutant males had an approximately three-fold decrease in TORC1 activity relative to control males while *wdr24*^*1*^ mutant females had a four-fold decrease in TORC1 activity relative to control females (Fig 2J and 2K). Taken together our data demonstrate that unlike the GATOR2 components Mio and Seh1, Wdr24 plays a central role in promoting TORC1 activity and growth in both germline and somatic tissues of *Drosophila* under standard culture conditions.

### *wdr24* mutants accumulate autolysosomes

**Fig 3:**
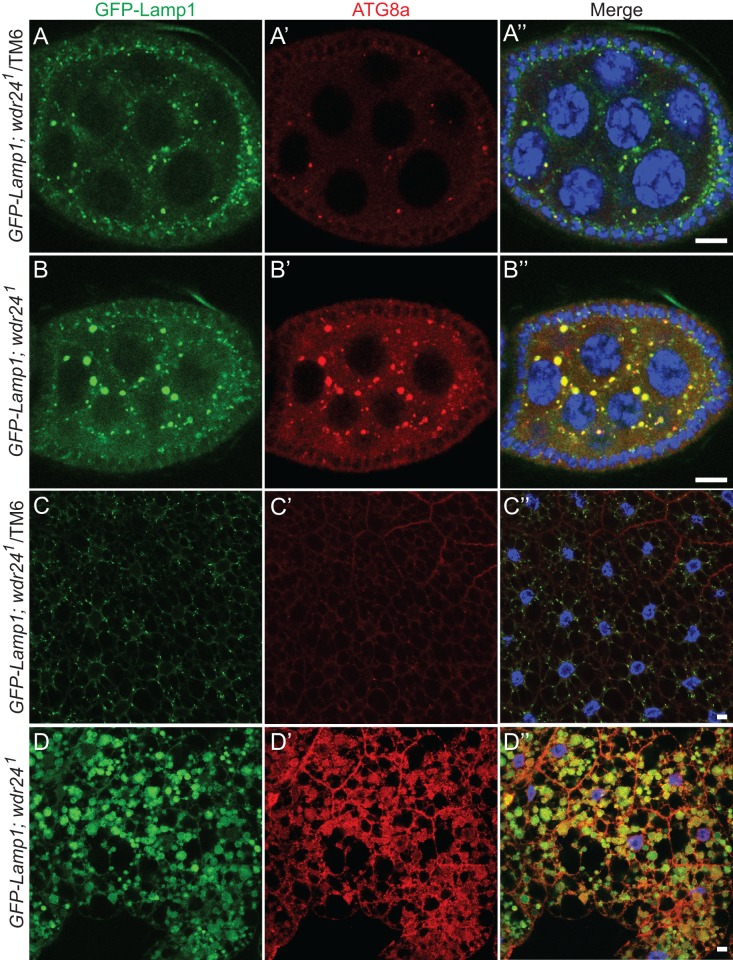
Wdr24 inhibits the accumulation of autolysosomes in the presence of amino acids. (A-B”) *wdr24* mutants accumulate autolysosomes in the female germline. Ovarioles from GFP-Lamp1/ CyO*; wdr24*^*1*^/*TM6* (A-A”) and GFP-Lamp1/ CyO*; wdr24*^*1*^ (B-B”) females stained with GFP, Atg8a antibodies and Hoechst. (C-D”) *wdr24*^*1*^ mutants accumulate autolysosomes in the larval fat body. Fat bodies from GFP-Lamp1/ CyO*; wdr24*^*1*^/*TM6* (C-C”) and GFP-Lamp1/ CyO*; wdr24*^*1*^(D-D”) third instar larvae stained with GFP, Atg8a antibodies and Hoechst. Size bar is 10μm.

TORC1 activity inhibits catabolic metabolism and autophagy [1]. Thus, we next examined if the low TORC1 activity observed in *wdr24*^*1*^ mutants resulted in the induction of autophagy in the absence of starvation. To assess the metabolic state of *wdr24*^*1*^ egg chambers, we followed GFP-Lamp1 and the autophagy marker Atg8a in ovaries from well-fed wild type and *wdr24*^*1*^ mutant females. Consistent with the activation of autophagy, egg chambers from well-fed *wdr24*^*1*^ mutant females are filled with Atg8a positive puncta that are also positive for the lysosomal marker GFP-Lamp1 (Fig 3A–3B”). This co-staining strongly suggests that these cytoplasmic puncta are autolysosomes. Thus, *wdr24*^*1*^ mutant egg chambers activate autophagy and accumulate autolysosomes in the female germ line independent of nutritional status. Next we examined the regulation of autophagy in somatic tissues. Under rich culture conditions, the larval fat bodies from wild-type and *seh1* null mutants contain a relatively small number of LysoTracker positive puncta (S4 Fig) [18]. In contrast, as is observed in *wdr24*^*1*^ mutant ovaries, fat bodies from well-fed *wdr24*^*1*^ larvae are filled with GFP-Lamp1 and Atg8a positive structures (Fig 3C–3D”). Importantly, this phenotype was rescued when GFP-tagged Wdr24 protein was expressed in the fat bodies of *wdr24*^*1*^ mutant larvae (S5 Fig). Taken together our data indicate that the Wdr24 component of the GATOR2 complex prevents the inappropriate accumulation of autolysosomes in both germline and somatic tissues of *Drosophila*.

**Fig 4:**
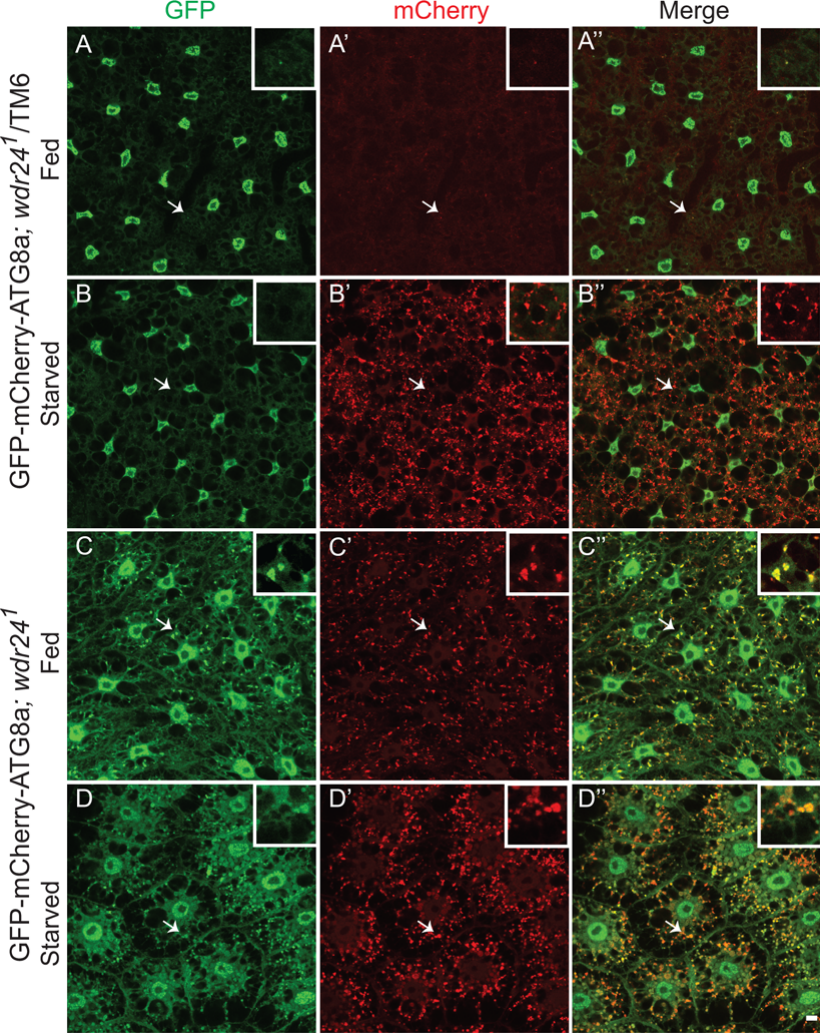
Wdr24 promotes autophagic flux. (A-B”) Live cell imaging of *Drosophila* fat bodies from Cg-Gal4/GFP-mCherry-Atg8a*; wdr24*^*1*^/*TM6* (control) third instar larvae cultured on standard fly medium (fed) (A-A”) or on PBS (starved) (B-B”). (C-D”) Live cell imaging of *Drosophila* fat bodies from Cg-Gal4/GFP-mCherry-Atg8a*; wdr24*^*1*^ (mutant) third instar larvae cultured on standard fly medium (fed) (C-C”) or on PBS (starved) (D-D”). Size bar is 10 μm.

In *Drosophila*, a tagged version of the autophagic marker Atg8a (GFP-mCherry-Atg8a) can be used to examine autophagic flux [29]. The double-tagged Atg8a protein is yellow (green merged with red) in autophagosomes, which are nonacidic structures but is red in autolysosomes due to the quenching of GFP fluorescence in acidic conditions [31]. To monitor autophagic flux we expressed GFP-mCherry-Atg8a protein in the fat body of wild-type and *wdr24*^*1*^ mutant larvae. In wild-type larvae starvation activates autophagy resulting in the accumulation of Atg8a positive puncta that are predominantly red, reflecting the accumulation of acidic autolysosomes (Fig 4A–4B”). In contrast, under both fed and starved conditions, the puncta in *wdr24*^*1*^ mutant fat bodies were yellow (Fig 4C–4D”). Similar yellow GFP-mCherry-Atg8a autolysosomes are observed in the fat body after starvation in knockdowns of subunits of the V-ATPase that is responsible for the acidification of lysosomes [32]. Thus, our data indicate that Wdr24 is required for autolysosome acidification and autophagic flux.

### Wdr24 opposes the activity of the GATOR1 complex in germline and somatic cells

**Fig 5:**
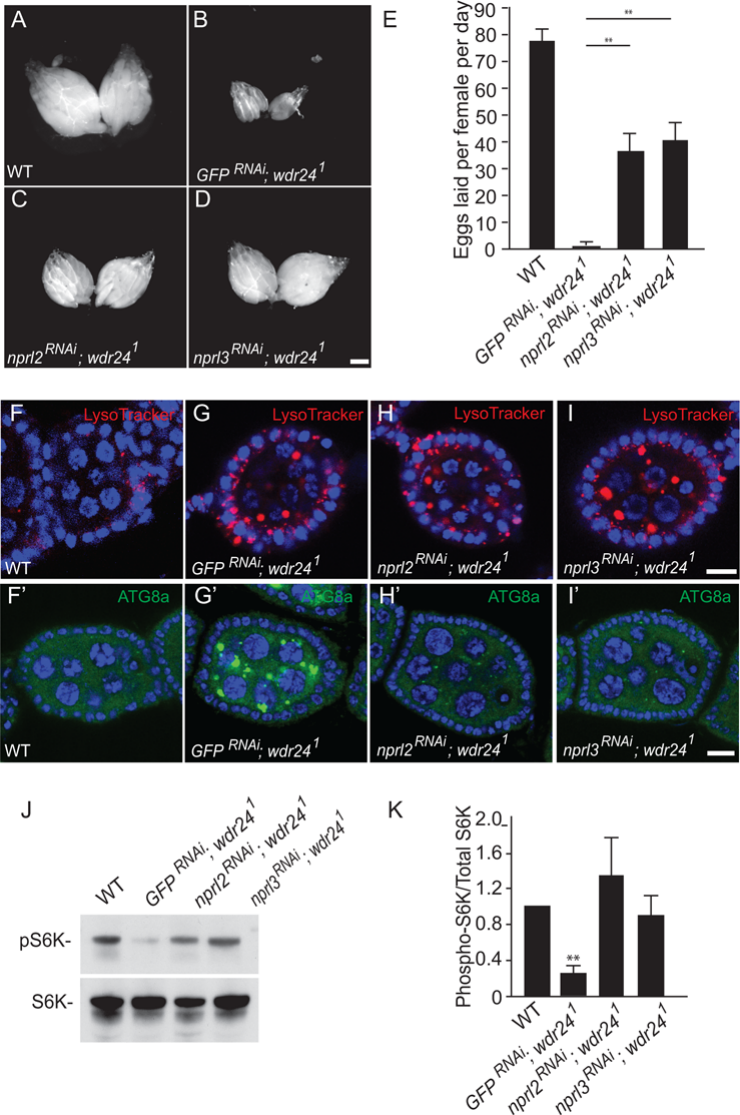
Germline depletions of *nprl2* and *nprl3* in *wdr24*^*1*^ mutant ovaries. (A-D) Dissected ovaries from wild type (WT) (A), *GFP*^*RNAi*^*; wdr24*^*1*^ (B), *nprl2*^*RNAi*^*; wdr24*^*1*^ (C) and *nprl3*^*RNAi*^*; wdr24*^*1*^ (D) females. *GFP*^*RNAi*^*; wdr24*^*1*^ was used as a negative control. Size bar is 100 μm. (E) Bar graph shows the number of eggs laid by WT, *GFP*^*RNAi*^*; wdr24*^*1*^, *nprl2*^*RNAi*^*; wdr24*^*1*^ *and nprl3*^*RNAi*^*; wdr24*^*1*^ females. Error bars represent the standard deviation for three independent experiments. ** p value < 0.01 (F-I’) Depleting *nprl2* and *nprl3* fails to rescue the LysoTracker accumulation phenotype in *wdr24*^*1*^ ovaries. Ovarioles from WT (F and F’) *GFP*^*RNAi*^*; wdr24*^*1*^ (G and G’), *nprl2*^*RNAi*^*; wdr24*^*1*^ (H and H’) and *nprl3*^*RNAi*^*; wdr24*^*1*^ (I and I’) females were stained with LysoTracker and Hoechst. Size bar is 10 μm. (J) Proteins isolated from WT, *GFP*^*RNAi*^*; wdr24*^*1*^, *nprl2*^*RNAi*^*; wdr24*^*1*^ *and nprl3*^*RNAi*^*; wdr24*^*1*^ ovaries were analyzed by Western blot probed with pS6K and S6K antibodies. (K) Quantification of phospho-S6K levels relative to the total S6K. Error bars represent the standard deviation for four independent experiments. ** p value < 0.01.

The GATOR2 components Mio and Seh1 promote TORC1 activation by opposing the activity of the GATOR1 complex [3,5,18]. Therefore, in order to determine whether the phenotypes observed in *wdr24* mutants are due to the unopposed TORC1 inhibitory activity of GATOR1, we examined the epistatic relationship between *wdr24* and the GATOR1 components *nprl2* and *nprl3*. First, we depleted *nprl2* and *nprl3* in the female germline of *wdr24*^*1*^ mutant females using RNAi. Notably, depleting *nprl2* or *nprl3* substantially rescued the *wdr24*^*1*^ ovarian phenotype, resulting in a dramatic increase in ovary size and a nearly six-fold increase in the number of eggs laid per female per day (Fig 5A–5E). In order to determine whether the reduced body size in *wdr24*^*1*^ mutant adults also reflects the unopposed TORC1 inhibitory activity of the GATOR1 complex, we used the ubiquitous GAL4 driver Hsp70-GAL4 to globally deplete the *nprl3* and *nprl2* transcript in the *wdr24*^*1*^ mutant background. Similar to our results from the germline, depleting *nprl3* and *nprl2* in somatic tissues significantly rescued the reduced body weight phenotype of *wdr24*^*1*^ mutants (S6 Fig). Thus, in *Drosophila* Wdr24 is required to oppose the activity of the GATOR1 complex in both germline and somatic tissues.

### Wdr24 regulates lysosome dynamics independent of TORC1 activity

As described above, *wdr24*^*1*^ mutants accumulate autolysosomes in the germline-derived nurse cells and oocytes of developing egg chambers as well as in the somatically derived cells of the larval fat body (Fig 3). One model to explain this phenotype, is that in *wdr24* mutants, the deregulation of the GATOR1 complex results in low TORC1 activity leading to the constitutive activation of catabolic metabolism and autophagy [18]. In this model, the accumulation of autolysosomes in *wdr24*^*1*^ mutant egg chambers is downstream of GATOR1 deregulation and low TORC1 activity. Surprisingly, we find that while depleting *nprl2* or *nprl3* in the germline of *wdr24*^*1*^ mutant females rescues the egg chamber growth defect, these egg chambers continue to accumulate a large number of LysoTracker positive puncta (Fig 5F–5I). The accumulation of these acidic puncta occur even though the levels of TORC1 activity in *nprl2*^*RNAi*^*; wdr24*^*1*^, and *nprl3*^*RNAi*^, *wdr24*^*1*^ovaries are similar or higher to those observed in wild-type ovaries (Fig 5J and 5K). Consistent with increased TORC1 activity, the acidic puncta in the *nprl2*^*RNAi*^*; wdr24*^*1*^, and *nprl3*^*RNAi*^, *wdr24*^*1*^ ovaries are not positive for the autophagic marker Atg8a indicating the autophagy pathway has not been activated (Fig 5F’–5I’). These data strongly suggest that the accumulation of autolysosomes in *wdr24*^*1*^ mutant ovaries is not solely the result of low TORC1 activity, but may reflect a second role for *wdr24* in the regulation of lysosome function and autophagic flux.

To confirm that Wdr24 affects lysosome dynamics independent of TORC1 activity, we knocked down *tuberous sclerosis 1* (*tsc1)* in the germline of *wdr24^1^* mutant females. The Tsc1/2 complex is a potent inhibitor TORC1 activity that functions independently of the GATOR1 complex [33,34]. In *Drosophila*, mutations in *tsc1* and *tsc2* (*gigas*) increase the baseline levels of TORC1 activity independent of nutrient status [35–38]. We found that similar to what was observed for depleting *nprl2* and *nprl3*, depleting *tsc1* in the *wdr24^1^* mutant background rescues the ovary growth deficit and results in TORC1 activity that is markedly higher than that observed in wild-type ovaries (S7A and S7B Fig). However, *tsc1^RNAi^; wdr24^1^* egg chambers continue to accumulate large numbers of LysoTracker positive puncta (S7C–S7E Fig). These data confirm that low TORC1 activity is not the cause of autolysosome accumulation in *wdr24* mutant ovaries.

**Fig 6:**
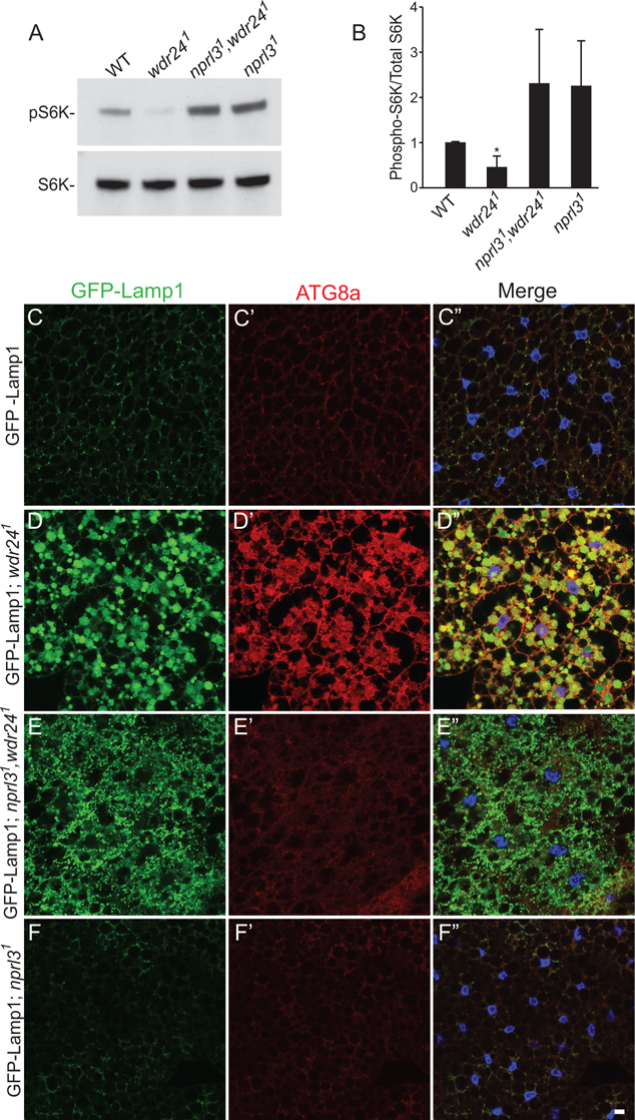
Wdr24 influences lysosome dynamics independent of TORC1 activity. (A) Proteins isolated from wild type (WT), *wdr24*^*1*^, *nprl3*^*1*^, *wdr24*^*1*^ and *nprl3*^*1*^ third instar larvae were analyzed by Western blot probed with pS6K and S6K antibodies. (B) Quantification of phospho-S6K levels relative to the total S6K. Error bars represent the standard deviation for three independent experiments. * p value < 0.05. (C-F’) Fat bodies from GFP-Lamp1/ CyO (C-C”), GFP-Lamp1/ CyO*; wdr24*^*1*^ (D-D”), GFP-Lamp1/ CyO*; wdr24*^*1*^, *nprl3*^*1*^ (E-E”) and GFP-Lamp1/ CyO*; nprl3*^*1*^ (F-F”) third instar larvae stained with GFP, Atg8a antibodies and Hoechst. Size bar is 10 μm.

Finally, we used epistasis analysis to formally test the model that *wdr24* regulates lysosome dynamics independent of the GATOR1 component *nprl3*. To accomplish this goal we generated a deletion allele of the GATOR1 component *nprl3*, that we named *nprl3^1^,* which removes 90% of the *nprl3* ORF (S8 Fig). A high percentage of *nrpl3^1^* single mutant females die as pupae or pharate adults. Thus, we focused our analysis on phenotypes observed in the larval fat body. First we assayed TORC1 activity by determining the phosphorylation status of the downstream TORC1 target S6 kinase. TORC1 activity was dramatically increased in larval fat bodies from *nprl3^1^* null mutants but was slightly decreased in larval fat bodies from *wdr24^1^* null mutants. In *wdr24^1^, nprl3^1^* double mutants, TORC1 activity was dramatically increased in the larval fat body relative to fat bodies from wild-type and *wdr24^1^* mutant larvae (Fig 6A and 6B). These data confirm that *nprl3* is epistatic to *wdr24* with respect to the regulation of TORC1 activity. However, although TORC1 activity is high, the fat bodies from *wdr24^1^, nprl3^1^* double-mutants accumulate large numbers of GFP-Lamp1 positive puncta. Importantly, these GFP-Lamp1 positive puncta are not observed in fat bodies from *nprl3^1^* single mutants (Fig 6C–6F). Thus, *wdr24* is epistatic to *nprl3* with regards to the accumulation of GFP-Lamp1 positive puncta. Taken together our data strongly suggest that the Wdr24 component of the GATOR2 complex has TORC1 dependent and independent functions.

Finally, GFP-Lamp1 positive puncta in *wdr24^1^* single mutants, that activate autophagy, are greatly enlarged relative to GFP-Lamp1 positive puncta that accumulate in *wdr24^1^, nprl3^1^* double mutants, which fail to activate autophagy (compare Fig 6D to 6E). These observations are consistent with the model that *wdr24* mutants fail to properly digest the contents of autolysosomes resulting in the accumulation of partial digested cellular components. This possibility is examined in greater detail below.

### Wdr24 regulates lysosome dynamics independent of autophagy

TORC1 inhibition activates the autophagy pathway initiating the formation of autophagosomes, which fuse with lysosomes to produce autolysosomes [2]. Thus, the accumulation of autolysosomes could be the result of a disruption in the regulation of autophagy or a disruption in the regulation of lysosome biogenesis and/or function. The high TORC1 activity observed in the fat bodies of *wdr24^1^, nprl3^1^* double-mutants should inhibit the activation of the autophagy pathway. Consistent with this idea, although *wdr24^1^, nprl3^1^* double-mutants have a large number of GFP-lamp1 positive puncta they have very few puncta that are positive for the autophagy marker Atg8a (Fig 6C’–6F’). Thus, the majority of the abnormal GFP-Lamp1 positive puncta found in *wdr24^1^, nprl3^1^* mutants represent late endosomes or lysosomes not autolysosomes. Similar observations were also made in *nprl2^RNAi^, wdr24^1^*, and *nprl3^RNAi^, wdr24^1^*ovaries (Fig 5F’–5I’). From these data we infer that *wdr24* regulates lysosome dynamics independent of the down-regulation of TORC1 activity and the activation of the autophagy.

In order to formally test the hypothesis that *wdr24* mutants accumulate abnormal lysosomes independent of the activation of the autophagy we generated *wdr24^1^* and *atg7^d14/d77^* double mutants. *atg7^d14^* and *atg7^d77^* are deletion alleles of *atg7* that function as null alleles [39,40]. In *Drosophila*, *atg7* is required for the activation of autophagy in response to starvation [41]. Therefore, if the autolysosome accumulation we observed in *wdr24^1^* mutant egg chambers requires the activation of autophagy, the LysoTracker positive puncta should be dramatically reduced or absent in *atg7^d14/d77^*, *wdr24^1^* double mutant egg chambers. Notably, however, we continue to observe a large number of LysoTracker puncta in *atg7^d14/d77^*, *wdr24^1^* double-mutant egg chambers (S9 Fig). Again, these puncta are not Atg8a positive, strongly suggesting they are late endosomes/lysosomes not autolysosomes. Taken together, our data demonstrate that *wdr24* regulates lysosome dynamics independent of the activation of autophagy.

### Wdr24 promotes lysosome acidification and autophagic flux in HeLa cells

**Fig 7:**
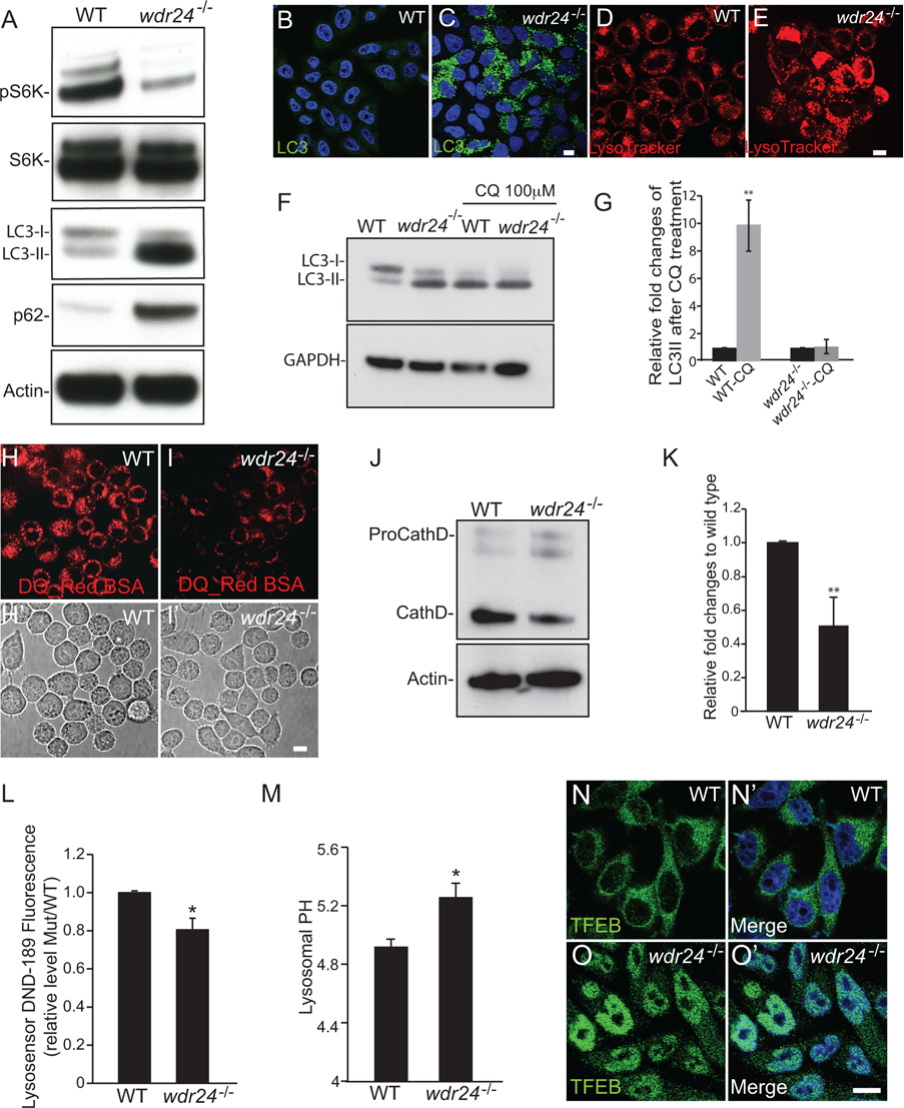
WDR24 regulates lysosomal acidification and autophagic flux in HeLa cells. (A) Proteins isolated from WT and *wdr24*^*-/-*^ HeLa cells were analyzed by Western blot probed with pS6K, S6K, LC3, p62 and actin antibodies. (B-E) Wild type (WT) (B and D) and *wdr24*^*-/-*^ (C and E) HeLa cells were stained with LC3 antibody, LysoTracker and DAPI. (F) Lysates from WT and *wdr24*^*-/-*^ HeLa cells treated or untreated with chloroquine were analyzed by Western blot probed with LC3 and GAPDH antibodies. (G) Quantification of relatively fold changes of LC3II level after chloroquine treatment. Error bars represent the standard deviation for three independent experiments. ** p < 0.01. (H and I) Confocal images show the fluorescent degradation products of the DQ-BSA in lysosomes in WT (H) and *wdr24*^*-/-*^ (I) HeLa cells. (H’ and I’) Bright field images of WT and *wdr24*^*-/-*^ HeLa cells in H and I respectively. (J) Western blot probed with antibodies against Cathepsin D. Actin was used as an internal control. (K) Quantification of cleaved Cathepsin D levels relative to actin is shown. Error bars represent the standard deviation for five independent experiments. ** p < 0.01. (L) Quantification of relatively fluorescent intensity from microplate reader measurement of Lysosensor DND-189 stained cells. Error bars represent the standard deviation for three independent experiments. * p< 0.05. (M) Quantification of lysosomal pH in lysosomes in WT and *wdr24*^*-/-*^ HeLa cells as determined by lysosensor Yellow/Blue DND-160 stained cells. Error bars represent the standard deviation for three independent experiments. * p< 0.05. (N-O’) Wild type (WT) (N, N’) and *wdr24*^*-/-*^ (O, O’) HeLa cells were stained with TFEB antibody and DAPI.

The WDR24 protein is conserved from yeast to mammals [17]. Thus, to investigate the cellular mechanism of lysosome/autolysosome dysfunction observed in the *wdr24* mutants in *Drosophila*, we knocked out the *WDR24* gene in human HeLa cells using CRISPR/CAS9 [42]. Specifically, we deleted the *WDR24* genomic region from 587–882 bp, which includes the start codon (S10A Fig). Western blot analysis showed that the *wdr24^-/-^* cell line did not express the WDR24 protein (S10B Fig). To confirm the role of WDR24 in TORC1 regulation, we analyzed the phosphorylation status of S6 kinase and 4E-BP1 in *wdr24^-/-^* cells. As expected, the *wdr24^-/-^* cell line had significantly reduced TORC1 activity (Fig 7A, S10C and S10D Fig). Moreover, the levels of S6 kinase phosphorylation were rescued when an HA tagged WDR24 protein was expressed in the *wdr24^-/-^* cells (S10E Fig). Thus, WDR24 functions to promote TORC1 activity in HeLa cells.

In addition to having decreased TORC1 kinase activity, *wdr24^-/-^* HeLa cells accumulate large numbers of autolysosomes, suggesting a conserved function for WDR24 in the regulation of autophagic flux in *Drosophila* and mammals (Fig 7B–7E). To better define the role of WDR24 we measured the levels of the autophagy protein LC3-II. Upon autophagy activation, the LC3-I protein is lipidated to generate LC3-II, which is hydrophilic and can integrate into autophagosomal membranes [43,44]. We found that LC3-II levels were dramatically increased in *wdr24^-/-^* cells relative to controls (Fig 7A). Increased LC3-II levels can be associated with either enhanced autophagosome synthesis or reduced autophagosome turnover due to diminished lysosomal degradation [44] To differentiate between these two mechanisms we measured p62 expression in *wdr24^-/-^* cells. p62 is a ubiquitin-binding protein that is degraded by autophagy; when autophagic flux is blocked, the degradation of p62 is reduced and the protein accumulates [45]. Conversely, if autophagic flux is increased, the degradation of the p62 protein is accelerated and thus the level of the protein decreases [46,47]. As predicted from our work in *Drosophila*, p62 levels are increased in *wdr24^-/-^* cells (Fig 7A and S11 Fig), indicating that autophagic flux requires the GATOR2 component WDR24. An additional assay to measure autophagic flux utilizes the lysosomal inhibitor chloroquine. In wild-type cells LC3 II levels are dramatically increased upon treatment with chloroquine (Fig 7F and 7G). This increase in LC3 II results from a block to autophagic flux due to decreased lysosomal activity. However, *wdr24^-/-^* mutant cells treated with chloroquine do not have increased LC3 II levels indicating that these mutants have diminished autophagic flux independent of chloroquine treatment (Fig 7F and 7G). Taken together these data strongly suggest that *wdr24^-/-^* cells exhibit reduced autophagic flux.

To further explore the proteolytic capabilities of lysosomes in *wdr24^-/-^* mutant cells, we used DQ BSA Bovine Serum Albumin (BSA) Red dequeching assay, a derivative of BSA labeled with BODIPY dyes that are strongly self-quenched. The proteolysis of the BSA molecule in lysosomes releases dequenched protein fragments, thus lysosomal proteolytic activity can be monitored by fluorescent intensity [48]. Notably, while *wdr24^-/-^* cells have a large increase in the number of autolysosomes there are fewer dequenched DQ-BSA-containing vesicles in *wdr24^-/-^* cells relative to wild-type cells (Fig 7H–7I’). These data indicate that *wdr24^-/-^* lysosomes have decreased proteolytic activity. As a further measure of lysosomal proteolytic activity we measured cleaved Cathepsin D levels by Western blot. Cathepsin D is a lysosomal protease that is cleaved in lysosomes into a mature enzyme [49]. We find that the levels of processed Cathepsin D are significantly reduced in *wdr24^-/-^* cells, again strongly suggesting that the lysosomes have diminished proteolytic activity (Fig 7J and 7K). However, it is possible that Wdr24 influences the trafficking of lysosomal enzymes, such as Cathepsin D, to lysosomes [50]. In order to test if Cathepsin D is properly localized to lysosomes in the *wdr24^-/-^* mutant cells, we stained mutant and wild type HeLa cells with antibodies against LAMP1 and Cathepsin D. Notably, these proteins strongly co-localize to lysosomes in both wild-type and *wdr24^-/-^* cells (S12 Fig). These data strongly suggest that trafficking of lysosomal enzymes to lysosomes is not dramatically diminished in the *wdr24^-/-^* HeLa cells.

Chloroquine blocks autophagic flux by increasing lysosomal pH [51]. Thus we reasoned that loss of Wdr24 protein might increase lysosomal pH. In order to test this possibility we used two different reagents, LysoSensor Green DND-189 and LysoSensor Yellow/Blue DND-160, to measure lysosome acidification. LysoSensor Green DND-189 fluorescent intensity increases in more acidic environments [51–53]. As shown in Fig 7L, *wdr24^-/-^* HeLa cells had lower fluorescent intensity, and thus higher lysosomal pH, relative to wild-type HeLa cells. Additionally, using LysoSensor Yellow/Blue DND-160, a ratiometric dye that can be used to measure the pH of acidic organelles, we found that the pH of lysosomes in *wdr24^-/-^* cells was increased to 5.25 relative to the pH of lysosomes in controls cells 4.92 (Fig 7M). Taken together these data support the model that the inhibition of autophagic flux observed in *wdr24^-/-^* knock out HeLa cells is due in part to the impairment of lysosomal acidification.

The transcription factor EB (TFEB) regulates lysosome biogenesis and function by promoting lysosomal gene expression [54–57]. Under non-starvation conditions, TFEB is phosphorylated by mTORC1 and is retained in the cytoplasm. When cells are starved, TFEB becomes dephosphorylated and is subsequently translocated into the nucleus to drive lysosomal and autophagic gene expression [54–56,58]. We find that the majority of TFEB is located in the nucleus of *wdr24^-/-^* cells indicating that TFEB has been activated (Fig 7N–7O’). This observation is consistent with the reduced TORC1 activity of *wdr24^-/-^* cells (Fig 7A). These data suggest that Wdr24 does not affect lysosome function by preventing the activation of TFEB.

Finally, consistent with the immunofluorescence and biochemical studies, Transmission Electron Microscopy (TEM) of *wdr24^-/-^* cells reveals a dramatic accumulation of autolysosome like structures that contain partially digested material (S13 Fig). Taken together our results provide strong evidence that in addition to regulating TORC1 activity the WDR24 protein promotes lysosome function and autophagic flux in mammalian cells.

### Additional GATOR2 components regulate lysosome function independent of TORC1 status

**Fig 8:**
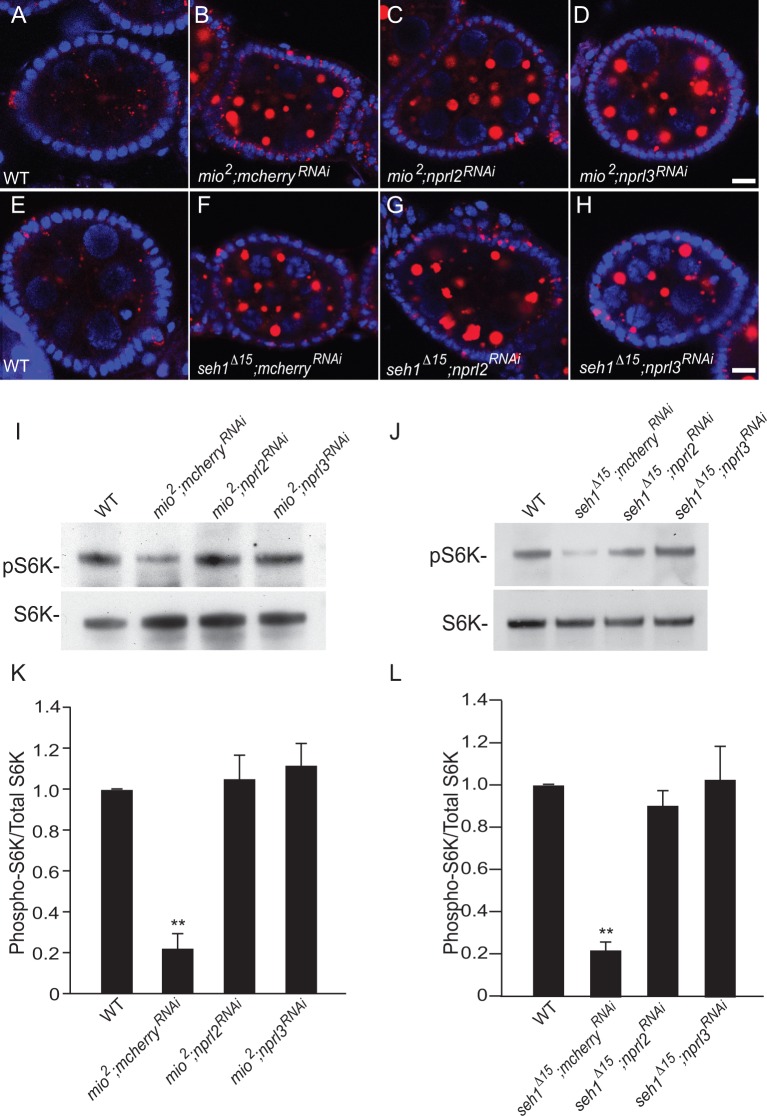
Mio and Seh1 influence lysosome dynamics independent of TORC1 activity. (A-H) Depleting *nprl2* and *nprl3* by germline specific driver Nanos-Gal4 fails to rescue the LysoTracker accumulation phenotype in *mio*^*2*^ and *seh1*^*Δ15*^ ovaries. Ovarioles from WT (A) *mio*^*2*^*; mCherry*^*RNAi*^ (B), *mio*^*2*^*; nprl2*^*RNAi*^ (C) and *mio*^*2*^*;nprl3*^*RNAi*^ (D) or from *WT* (E), *seh1*^*Δ15*^*; mCherry*^*RNAi*^ (F), *seh1*^*Δ15*^*; nprl2*^*RNAi*^ (G) and *seh1*^*Δ15*^*;nprl3*^*RNAi*^ (H) females were stained with LysoTracker and Hoechst. Size bar is 10 μm. (I and J) Proteins isolated from WT, *mio*^*2*^*; mCherry*^*RNAi*^, *mio*^*2*^*; nprl2*^*RNAi*^, *mio*^*2*^*;nprl3*^*RNAi*^ ovaries, or protein isolated from *WT*, *seh1*^*Δ15*^*; mCherry*^*RNAi*^, *seh1*^*Δ15*^*; nprl2*^*RNAi*^ and *seh1*^*Δ15*^*;nprl3*^*RNAi*^ ovaries were analyzed by Western blot probed with pS6K and S6K antibodies. (K and L) Quantification of phospho-S6K levels relative to the total S6K. Error bars represent the standard deviation for three independent experiments. ** p value < 0.01.

Finally, an important question remained. Does Wdr24 regulate lysosome function in the context of the GATOR2 complex or does Wdr24 function in an alternative complex to regulate lysosome dynamics? To address this question we examined if other GATOR2 complex components regulate lysosomal dynamics independent of their role in the regulation of TORC1 activity. For these experiments we returned to *Drosophila*. Specifically, we depleted the GATOR1 components *nprl2* and *nprl3* in the *mio*^2^and *seh1^Δ15^* mutant backgrounds. As was previously reported, we found that *nprl2* and *nprl3* depletions rescue the small ovary and fertility deficits of *mio*^2^and *seh1^Δ15^* mutants [18]. Importantly, however, although we found that the *nprl2* and *nprl3* depletions rescued the low TORC1 activity of *mio*^2^ and *seh1^Δ15^* ovaries, the depletions failed to rescue the accumulation of lysotracker positive puncta (Fig 8). From these data we infer that multiple components of the GATOR2 complex function in the TORC1 independent regulation of lysosomal function and autophagic flux in *Drosophila*. However, we note that we have not shown that all components of GATOR2 complex function in the regulation of lysosomes.

## Discussion

**Fig 9:**
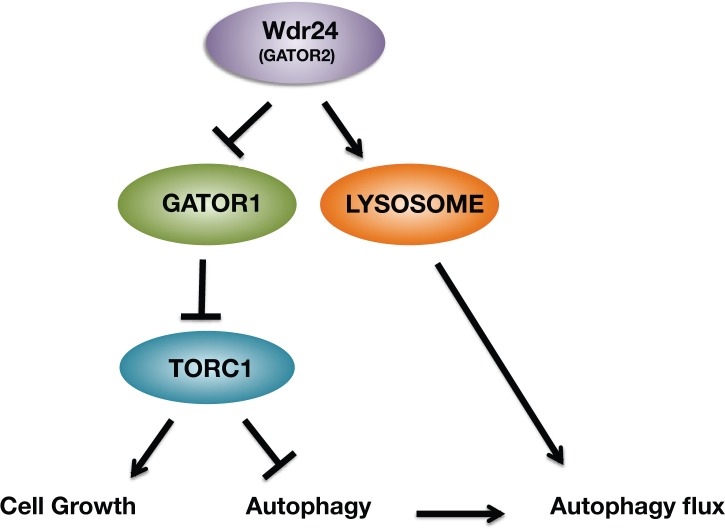
A dual role for the GATOR2 component Wdr24 in the regulation of TORC1 activity and lysosome function. Wdr24 promotes TORC1 activation by opposing the activity of the GATOR1 complex. Additionally, independent of TORC1 status, Wdr24 promotes lysosome acidification, which is required for autophagic flux.

Here we describe a dual role for the GATOR2 component Wdr24 in the regulation of TORC1 activity and lysosome dynamics. We demonstrate that Wdr24 is a critical effector of the GATOR2 complex that promotes TORC1 activity in both germline and somatic tissues. This lies in contrast to the GATOR2 components Mio and Seh1, which have a limited role in the regulation of TORC1 activity in many cell types [27,28]. Surprisingly, we identify a second function of Wdr24 that is independent of TORC1 status, the regulation of lysosome acidification and autophagic flux (Fig 9). Taken together our data support the model that the GATOR2 complex regulates both the response to amino acid starvation and lysosome function.

### Wdr24 is a critical effector of the GATOR2 complex

Whole animal studies often reveal tissue-specific and/or metabolic requirements for genes that are not readily observed in cell culture. In mammalian and *Drosophila* tissue culture cells, RNAi based depletions of the GATOR2 components Mio, Seh1, Wdr59, and Wdr24 result in decreased TORC1 activity in return to growth assays [3,18]. These data have resulted in the model that all components of the GATOR2 complex are generally required for TORC1 activation (3). However, the characterization of *mio* and *seh1* null mutants in *Drosophila*, demonstrated that Mio and Seh1 are critical for the activation of TORC1 and inhibition of autophagy in the female germ line, but play a relatively small role in the regulation of TORC1 activity and autophagy in somatic tissues under standard culture conditions [18,27,28]. Thus, the requirement for at least a subset of GATOR2 complex components is tissue and/or context specific.

Here we report that the GATOR2 component Wdr24 is required for the full activation of TORC1 in both germline and somatic cells of *Drosophila*. Consistent with the global down-regulation of TORC1 activity in the absence of Wdr24, we find that *wdr24* mutant adults are notably smaller than controls and are female sterile. Depleting the GATOR1 components *nprl2* and *nprl3* in the *wdr24* mutant background rescued the low TORC1 activity, growth defects, and female sterility of *wdr24* mutants. Thus, the GATOR2 component Wdr24 is required to oppose GATOR1 activity in both germline and somatic cells of *Drosophila*. From these results we propose that Wdr24 is a key effector of the GATOR2 complex required for the full activation of TORC1 in most cell types.

There are several potential models to explain the differential requirement for individual GATOR2 proteins in *Drosophila*. First, there may be tissue specific requirements for individual GATOR2 subunits. In this model the different phenotypes observed in the *seh1* and *mio* versus *wdr24* mutants reflects a qualitative difference in the requirement for these proteins in different tissues. However, we favor an alternative model in which Wdr24 is the core effector of GATOR2 activity, with Mio and Seh1 functioning primarily as positive regulators of GATOR2 activity. In this second model, the differential phenotypes observed in the *seh1* and *mio* versus *wdr24* mutants reflects a quantitative difference in the requirement for GATOR2 activity in different tissues. The distinction between these two models awaits the identification of the molecular mechanism of Wdr24 and GATOR2 action.

### Wdr24 regulates lysosome function independent of TORC1 status and autophagy

We have identified a novel TORC1 independent role for Wdr24 in the regulation of lysosome dynamics and function. In *wdr24* mutants, the down-regulation of TORC1 activity and the accumulation of autolysosomes occur independent of nutrient status. Our initial hypothesis was that in the absence of the GATOR2 component Wdr24, the deregulation of the GATOR1 complex results in low TORC1 activity, triggering the constitutive activation of autophagy and the accumulation of autolysosomes. Surprisingly, however, our epistasis analysis determined that the accumulation of lysosomes could be decoupled from both the chronic inhibition of TORC1 activity and the activation of autophagy. Raising TORC1 activity in the *wdr24* mutant background, by depleting either components of the GATOR1 or TSC complex, failed to rescue the accumulation of abnormal lysosomal structures. Notably, we determined that two additional members of the GATOR2 complex, Mio and Seh1, also regulate lysosomal behavior independent of both GATOR1 and the down-regulation of TORC1 activity. From these data we infer that multiple components of the GATOR2 complex have a TORC1 independent role in the regulation of lysosomes.

An increased number of autolysosomes is often associated with reduced autophagic flux due to diminished lysosomal degradation [44]. Consistent with reduced autophagic flux, in *Drosophila wdr24^-/-^* mutants accumulated enlarged autolysosomes filled with undegraded material. Moreover, lysosomes in the *wdr24^-/-^* mutants failed to quench the GFP fluorescence of a GFP-mCherry-Atg8a protein. These phenotypes are consistent with decreased lysosomal pH and degradative capacity [44]. In order to examine in detail the role of Wdr24 in the regulation of lysosome function we generated a *wdr24^-/-^* knockout HeLa cell line that recapitulated the phenotypes observed in *Drosophila wdr24^-/-^* mutants. Specifically, *wdr24^-/-^* HeLa cells had have decreased TORC1 activity and accumulate a large number of autolysosomes. Using multiple assays we determined that *wdr24^-/-^* lysosomes had reduced degradative capacity and autophagic flux and thus accumulate proteins that are normally degraded by lysosomal enzymes such as p62, LC3II and Cathepsin D. Additionally, we determined that *wdr24^-/-^* lysosomes have increased pH relative to wild-type cells, again consistent with reduced lysosomal function. Taken together these data confirm that Wdr24 plays a key role in the regulation of lysosomal activity.

Here we show that components of the GATOR2 complex function in the regulation of TORC1 activity and in the TORC1 independent regulation of lysosomal dynamics and autophagic flux. These two functions suggest that the GATOR2 complex may regulate cellular homeostasis by coordinating TORC1 activity with the dynamic regulation of lysosomes during periods of nutrient stress. Intriguingly, several recent reports describe a very similar dual function for the RagA/B GTPases in both mice and zebrafish [59,60]. RagA/B play a critical role in the activation of TORC1 in the presence of amino acids [61,62]. Surprisingly, however, TORC1 activity was not found to be significantly decreased in cardiomyocytes of RagA/B knockout mice (56). Nevertheless, the RagA/B mutant cardiomyocytes have decreased autophagic flux and reduced lysosome acidification. From their data, the authors conclude that the RagA/B GTPases regulate lysosomal function independent of their role in the regulation of TORC1 activation in some cell types [59]. Similarly, RagA is required for proper lysosome function and phagocytic flux in microglia [60]. Notably, Mio, a component of the GATOR2 complex is found associated with RagA [3]. Thus, in the future it will be important to determine if components of the GATOR2 complex function in a common pathway with the Rag GTPases to regulate lysosomal function.

In *Saccharomyces cerevisiae* single mutants of *wdr24/sea2* and *wdr59/sea3* do not exhibit defects in TORC1 regulation but do have defects in vacuolar structure [4,5,63]. Moreover, several recently identified genes that regulate the GATOR2-GATOR1-TORC1 pathway in response to amino acid limitation are restricted to metazoans [20–25]. These data make it tempting to speculate that the ancestral function of the GATOR2 complex maybe the regulation of lysosome/vacuole function and autophagic flux. Indeed, the finding that GATOR2 components regulate lysosome dynamics is particularly intriguing in light of the observation that GATOR2 complex is comprised of proteins with characteristics of coatomer proteins and membrane tethering complexes [17]. Notably, the GATOR2 complex components Mio, Seh1 and Wdr24 localize to lysosomes and autolysosomes [18]. Similarly, these proteins associate with the vacuolar membrane in budding yeast [17]. Thus, going forward it will be important to examine if the GATOR2 complex acts directly on lysosomal membranes to regulate their structure and/or function. More broadly, future studies on the diverse roles of the SEACAT/GATOR2 complex will further our understanding of the complex relationship between cellular metabolism and the regulation of endomembrane dynamics in both development and disease.

## Materials and Methods

### *Drosophila* strains and genetics

MTD-GAL4, UASp-mCherry-Atg8a *(y^1^ w^1118^; P{UASp-mCherry-Atg8a}2; Dr^1^/TM3, Ser^1^)*, UASp-GFP-mCherry- Atg8a (*y^1^ w^1118^; P{UASp-GFP-mCherry-Atg8a}2, wdr24^1^* (*CG7609^1^*), UAS-Tsc1 RNAi (y^1^ sc* v^1^; P{TRiP.GL00012}attP2), HS-FLP; Ubi-GFP FRT82B/TM3 and Df(3R)BSC547 lines were obtained from the Bloomington Stock Center. The *mio^1^*, *mio^2^*, *seh1^Δ15^*, *nprl ^RNAi^* and *nprl3 ^RNAi^* lines were described previously [18,64]. GFP-Lamp1 *(w^1118^; P{W+, Tub>GFP-Lamp1}1/*CyO; TM6b*, Hu boss^1^/Sb boss^1^)* was kindly provided by Helmut Kramer (UT Southwestern). Nanos-Gal4 *(P{NANOS GAL4 VP-16}*, *yw;* D/TM3, Ser, Sb*)* was kindly provided by Sharon Bickel (Dartmouth College)*. atg7^d77^/*Cyo-GFP *and atg7^d14^/*CyO-GFP were kindly provided by Thomas P. Neufeld (University of Minnesota) [41]. All fly stocks were maintained on JAZZ-mix *Drosophila* food (Fisher Scientific) at 25℃.

### Generation of transgenic lines

The *wdr24* coding region was cloned into a pENTR-1A vector (Invitrogen). The *mCherry* coding region was inserted into pENTR-wdr24 plasmid. The pENTR-*wdr24* and pENTR-*wdr24-mCherry* plasmids were recombined with pPGW vector (DGRC) and pPFHW vector (DGRC) separately and to generate UASp-*GFP*-*wdr24 and* UASp*-wdr24-mCherry* using Gateway LR Clonase II Enzyme (Invitrogen). UASp-*GFP-wdr24* and UASP-*wdr24-mCherry* plasmids were used to generate transgenic lines (Best gene Inc). All primers used for PCR amplification are listed in S2 Table.

### Tandem affinity purification and mass spectrometry

Ovaries from the transgenic flies that stably express Mio proteins tagged with FLAG and HA were homogenized in 50 mM Tris-HCl (pH 7.4), 300 mM NaCl, 5 mM EDTA, and 1% Triton X-100 supplemented with proteinase inhibitor cocktail (Roche) [27]. Cell lysates were cleared by centrifugation at 15,000 × *g* for 15 min and Proteins were purified using FLAG-HA tandem affinity purification kit (Sigma) Proteins were precipitated with 10% trichloroacetic acid (TCA), washed with acetone, air-dried, and analyzed by liquid chromatography (LC)/MS at the Taplin MS facility (Harvard Medical School).

### S2 cell transfection, immunoprecipitation (IP)

S2 cell transfection and immunoprecipitation were performed as previously described [18]. 2 μg GFP antibody (Roche), 20 μl protein G agarose (Millipore) and 10 μl protein A agarose (Roche) were used for each experiment.

### Western blot analysis

Whole flies or HeLa cells were lysed in RIPA buffer containing complete protease inhibitors and phosphatase inhibitors (Roche). Western blots were performed as described previously [18]. Antibodies were used at the following concentrations: rabbit anti-P-S6K T398 at 1:1000 (Cell Signaling), guinea pig anti-S6K at 1:10,000 (24), mouse anti-actin at 1:10,000 (Abcam), rabbit anti-LC3A/B at 1:1000 (Cell Signaling), rabbit anti-P-S6K T389 at 1:1000 (Cell Signaling), rabbit anti-S6K at 1:1000 (Cell Signaling), rabbit anti-GAPDH at 1:3000 (Cell Signaling), rabbit anti-P-4E-BP1at 1:1000 (Cell Signaling), rabbit anti-4E-BP1 at 1:1000 (Cell Signaling), rabbit anti-GFP at 1:500 (Cell Signaling), rabbit anti-SQSTM1/p62 at 1:500 (Cell Signaling), Mouse anti- SQSTM1/p62 at 1:1000 (Novus Biologicals), rabbit anti-WDR24 at 1:1000 (Novus Biologicals) and goat anti-Cathepsin D at 1:500 (Santa Cruz). The band intensity was quantified using Image J analysis tool (NIH).

### Immunofluorescence and live cell imaging

Immunofluorescence was performed as described [65], using the following antibodies: goat anti-GFP FITC conjugated (1:400, Abcam), rabbit anti-LC3A/B (1:1000, Cell Signaling), mouse anti- SQSTM1/p62 (1:100, Novus Biological), rabbit anti-TFEB (1:100, Cell Signaling), rabbit anti-CathD (1:100, Calbiochem) and rabbit anti-Atg8a (1:200, Abcam). Anti-rabbit and anti-mouse Alexa Fluor secondary antibodies (Invitrogen) were used at 1:1000. Nuclei were visualized by staining the DNA with DAPI or Hoechst 33324 (Invitrogen). Images were acquired using a Leica TCS SP5 confocal microscope. Live cell images were obtained as previously described [18].

### Clonal analysis

To generate *wdr24^1^* homozygous clones, HS-FLP; Ubi-GFP FRT82B/*wdr24^1^* FRT82B third instar larvae were heat shocked for 1hr in a 37°C water bath two times per day. The adult females were collected and aged for 5 to 7 days. Fat bodies or ovaries from adult flies were dissected and stained with GFP antibody (Abcam) and Hoechst 33324 (Invitrogen). Homozygous *wdr24^1^* clones were marked by the absence of GFP expression.

### Generation of CRISPR/Cas9 knock out flies

To generate knock out *nprl3* flies, guide RNAs (gRNA) that target *nprl3* were designed using the online CRIPR design tool (http://crispr.mit.edu/). To make the deletion mutants, two different gRNAs were cloned into pBFv-U6.2B as previously described [66]. pBFv-U6.2B-*nprl3* plasmids were injected into y[1], vas-Cas9, w^[1118]^ embryos. All the oligonucleotides used for cloning and screening are listed in S2 Table.

### Generation of CRISPR/Cas9 knock out cells

To generate knock out *WDR24* HeLa cells, two pairs of oligonucleotide encoding the guide RNAs were cloned into px330 vector [42]. On day one, 50,000 cells were seeded into 24-well plate. Each well was transfected with a total of 1 μg of a pair of *px330* guide constructs. The third day, cells were pooled into 10 cm dishes with 200 cells each dish. After 10 days, all the clones were collected and seeded into 24 well plates. Cells were grown and expanded, and the positive colonies were identified by PCR and sequencing. All primers used for PCR amplification are listed in S1 Table.

### HeLa cell culture, transfection and DQ BSA Red assay

HeLa cells and knock out cells were cultured at 37°C, 5% CO2 in DMEM medium (Life Technology) supplemented with 10% fetal bovine serum (FBS, Life Technology), 50 U/ml penicillin and 50 μg/ml streptomycin. Plasmid transfection was performed using Lipofectamine 2000 (Life Technologies). HAWDR24 pRK5 (Plasmid #46335) was obtained from Addgene. For the DQ BSA Red (Life Technology) assay, Cells were incubated for 4 h with DQ BSA (10 μg/ml) and then washed twice with HBSS. Subsequently the cells were incubated in the starvation medium (HBSS, starvation media; Life Technology) to induce autophagy. After 1 hour the DQ BSA fluorescence was detected using a Leica TCS SP5 confocal microscope.

### Transmission electron microscopy

Cells grown on coverslips were fixed in 2% (wt/vol) formaldehyde and 2% (wt/vol) glutaraldehyde in 0.1 M sodium cacodylate (pH 7.4), postfixed in 1% aqueous OsO4, and stained en bloc with 2% (wt/vol) uranyl acetate. Upon dehydration and embedding in EMBed-812 (EM Science, Horsham, PA), the coverslips were removed by hydrofluoric acid, cells were thin-sectioned parallel to the glass, and sections were stained with uranyl acetate. The samples were examined on an FEI Tecnai 20 transmission electron microscope operated at 80 kV, and images were recorded on a Gatan Ultrascan CCD camera.

### HeLa cell lysosomal pH measurement

Lysosomal pH measurements were performed as describe [67]. In brief, cells were stained with 1μM LysoSensor Green DND-189 in DMED regular medium for 20 min at 37°C, 5% CO2. Subsequently, cells were washed with PBS twice and analyzed by a microplate reader (485/530 nm) in triplicate. Lysosomal pH quantification was performed using LysoSensor Yellow/Blue DND-160. Cells were labeled with 1μM LysoSensor Yellow/Blue DND-160 for 30 min at 37°C, 5% CO2 in DMEM medium and then washed twice with PBS. To generate a calibration curve cells were treated for 10 min with 10μM monensin and 10μM nigericin in 25mM MES calibration buffer, pH 3.5–6.0, containing 5 mM NaCl, 115 mM KCl, and 1.2 mM MgSO. The fluorescence was measured with a microplate reader (340/440 nm and 380/530 nm) at 37°C. These experiments were performed in triplicate.

## Supporting Information

S1 Fig Generation of *wdr24*^*1*^ deletion mutant. (A) Schematic map shows the *wdr24*^*1*^ deletion. Dashed line marks the deletion position. (B) RT-PCR demonstrates that *wdr24* mutants do not produce full length mRNA. Integrator subunit 11 (*intS11)* was used as the internal control in order to demonstrate that the deletion only affects *wdr24* gene expression but not the neighboring gene *intS11*. (C-D) Expression of GFP-Wdr24 using the germline specific Nanos-Gal4 driver rescues the ovarian and fertility phenotypes of *wdr24* mutants. (C) Dissected ovaries from control *wdr24*^*1*^/*TM6*, *wdr24*^*1*^ and Nanos-Gal4; GFP-Wdr24; *wdr24*^*1*^ females. Size bar is 100 μm. (D) Bar graph shows the number of eggs laid by Nanos-Gal4; *wdr24*^*1*^*/TM6*, Nanos-Gal4; *wdr24*^*1*^ and Nanos-Gal4; GFP-Wdr24; *wdr24*^*1*^ females. Error bars represent the standard deviation for three independent experiments. ** p value < 0.01.
(TIF)

S2 Fig Wdr24 is not required for the maintenance of the oocyte fate. Wdr24 is not required for the maintenance of the oocyte fate. Ovaries stained with DAPI (DNA, blue) and Orb antibody (oocyte marker, red). (A) *wdr24*^*1*^/*TM6* (B) *wdr24*^*1*^ (C) *mio*^*1*^*/Df*. Note that the *wdr24*^*1*^/*TM6* and *wdr24*^*1*^ mutant egg chambers all contain ooctyes (white arrow), but multiple *mio*^*1*^*/Df* egg chambers have no apparent oocyte (white arrowhead). Size bar is 10 μm.
(TIF)

S3 Fig Expression of GFP-Wdr24 using the Ubi-Gal4 driver rescues the body weight phenotype of *wdr24* mutants. Bar graph shows that overexpression of GFP-Wdr24 using the Ubi-Gal4 driver in *wdr24*^*1*^ mutant background significantly increases body weight. ** p value < 0.01. n.s. indicates not significant.
(TIF)

S4 Fig *seh1* mutants do not accumulate large numbers of autolysosomes in somatic tissues and have a normal body weight. Fat bodies form *seh1*^*Δ15*^/*CyO-GFP* (A) and *seh1*^*Δ15*^ (B) third instar larvae stained with LysoTracker and Hoechst. Size bar is 10 μm. (C) Quantification of body weights of *seh1*^*Δ15*^/*SM6a* and *seh1*^*Δ15*^/*Df* adult males. Error bars represent the standard deviation for three sets of experiments (8 male flies per group). n.s. indicates not significant.
(TIF)

S5 Fig Expression of GFP-Wdr24 rescues the LysoTracker accumulation defect in *wdr24* mutants. (A-C’) Expression of GFP-Wdr24 using the Cg-Gal4 fat body driver in *wdr24*^*1*^ mutant background rescues the LysoTracker accumulation phenotype. Fat bodies from GFP-Wdr24*; wdr24*^*1*^ /*TM6* (A and A’), *wdr24*^*1*^ (B and B’) and GFP-Wdr24*; wdr24*^*1*^ (C and C’) third instar larvae were stained with LysoTracker and Hoechst. Size bar is 10 μm.
(TIF)

S6 Fig Wdr24 opposes the GATOR1 complex to promote growth in *Drosophila*. Bar graph shows that depleting *nprl2* and *nprl3* in the *wdr24*^*1*^ mutant background results in an increased body weight. *GFP*^*RNAi*^*; wdr24*^*1*^ served as a negative control. * p value < 0.05.
(TIF)

S7 Fig Tsc1 depletions result in increased TORC1 activity but do not prevent the inappropriate accumulation of lysosomes in *wdr24* mutant ovaries. (A) Proteins isolated from WT, *GFP*^*RNAi*^*; wdr24*^*1*^ ovaries and *tsc1*^*RNAi*^*; wdr24*^*1*^ were analyzed by Western blot probed with pS6K and S6K antibodies. (B) Quantification of phospho-S6K levels relative to the total S6K. Error bars represent the standard deviation for three independent experiments. ** p value < 0.01. (C-E) Depleting *tsc1* fails to rescue the lysosomal phenotype in *wdr24*^*1*^ ovaries. Ovarioles from WT (C) *GFP*^*RNAi*^*; wdr24*^*1*^ (D) *and tsc1*^*RNAi*^*; wdr24*^*1*^ (E) females were stained with LysoTracker and Hoechst. Size bar is 10 μm.
(TIF)

S8 Fig Generation of *nprl3* gene deletion. Schematic map shows the deletion end points of the *nprl3*^*1*^ allele. Dashed line marks the deletion position. N-terminal break point starts at 61th amino acid from start codon. The deletion causes frame shift and generates a new stop codon after 5 amino acids from the C-terminal break point.
(TIF)

S9 Fig Wdr24 regulates lysosome organization independent of autophagy. (A-D’) Ovarioles from *wdr24*^*1*^/*TM6* (A, A’), *wdr24*^*1*^ (B, B’), *atg7*^*d14/d77*^ (C, C’) and *atg7*^*d14/d77*^*; wdr24*^*1*^ (D, D’) females stained with LysoTracker, anti-Atg8a and Hoechst or DAPI. Note that *atg7;wdr24* double-mutant ovaries accumulate lysotracker positive puncta that are not positive for the autophagy marker ATG8a. Size bar is 10 μm.
(TIF)

S10 Fig Generation of a *WDR24* knock out HeLa cell line. (A) Schematic map shows the position of *wdr24*^*-/-*^ deletion. (B) Western blot of wild type (WT) and *wdr24*^*-/-*^ probed with WDR24 and actin antibodies. Note that the *wdr24*^*-/-*^ mutant cells do not express the WDR24 protein. (C) Western blot of wild type (WT) and *wdr24*^*-/-*^ probed with phospho-4E-BP and 4E-BP antibodies. Note that the *wdr24*^*-/-*^ mutant cells have lower phosphor-4E-BP level suggesting a decrease of mTORC1 activity. This experiment has been done in triplicates. (D) Quantification of phospho-4E-BP levels relative to the total 4E-BP. Error bars represent the standard deviation for three independent experiments. * p value < 0.05. (E) Western blot of cell lysates from WT, *wdr24*^*-/-*^ and *wdr24*^*-/-*^ HA-WDR24 rescued cells probed with antibodies against pS6K and S6K. Note that the overexpression HA tagged WDR24 protein in *wdr24*^*-/-*^ mutants increases mTORC1 activity as indicated by pS6K levels.
(TIF)

S11 Fig *wdr24*^*-/-*^ knock out HeLa cells accumulate p62. (A and B) Wild type (WT) (A) and *wdr24*^*-/-*^ (B) HeLa cells were stained with p62 antibody and DAPI. Size bar is 10 μm.
(TIF)

S12 Fig Cathepsin D trafficking is not affected in *wdr24*^*-/-*^ knock out HeLa cells. (A–B”) Wild type (WT) (A-A”) and *wdr24*^*-/-*^ (B-B”) HeLa cells were stained with Cathepsin D and LAMP1 antibodies. Size bar is 10 μm.
(TIF)

S13 Fig TEM images of autolysosomes in in *wdr24*^*-/-*^ knock out HeLa cells. (A-B’) TEM images of lysosomes or autolysosomes from WT (A) and *wdr24*^*-/-*^ (B) cells. Autolysosomes are shown at higher magnification for both WT (A′) and *wdr24*^*-/-*^ (B′) mutant HeLa cells. Yellow arrows mark autolysosomes. Size bar is 1 μm.
(TIF)

S1 Table Identification of the GATOR complex in *Drosophila*. (DOCX)

S2 Table Primer list. (DOCX)
