# Supplementary figures and images for: The GATOR2 Component Wdr24 Regulates TORC1 Activity and Lysosome Function
Source: PLoS Genet. 2016 May 11;12(5):e1006036. doi: 10.1371/journal.pgen.1006036 (PMC4864241; doi:10.1371/journal.pgen.1006036)

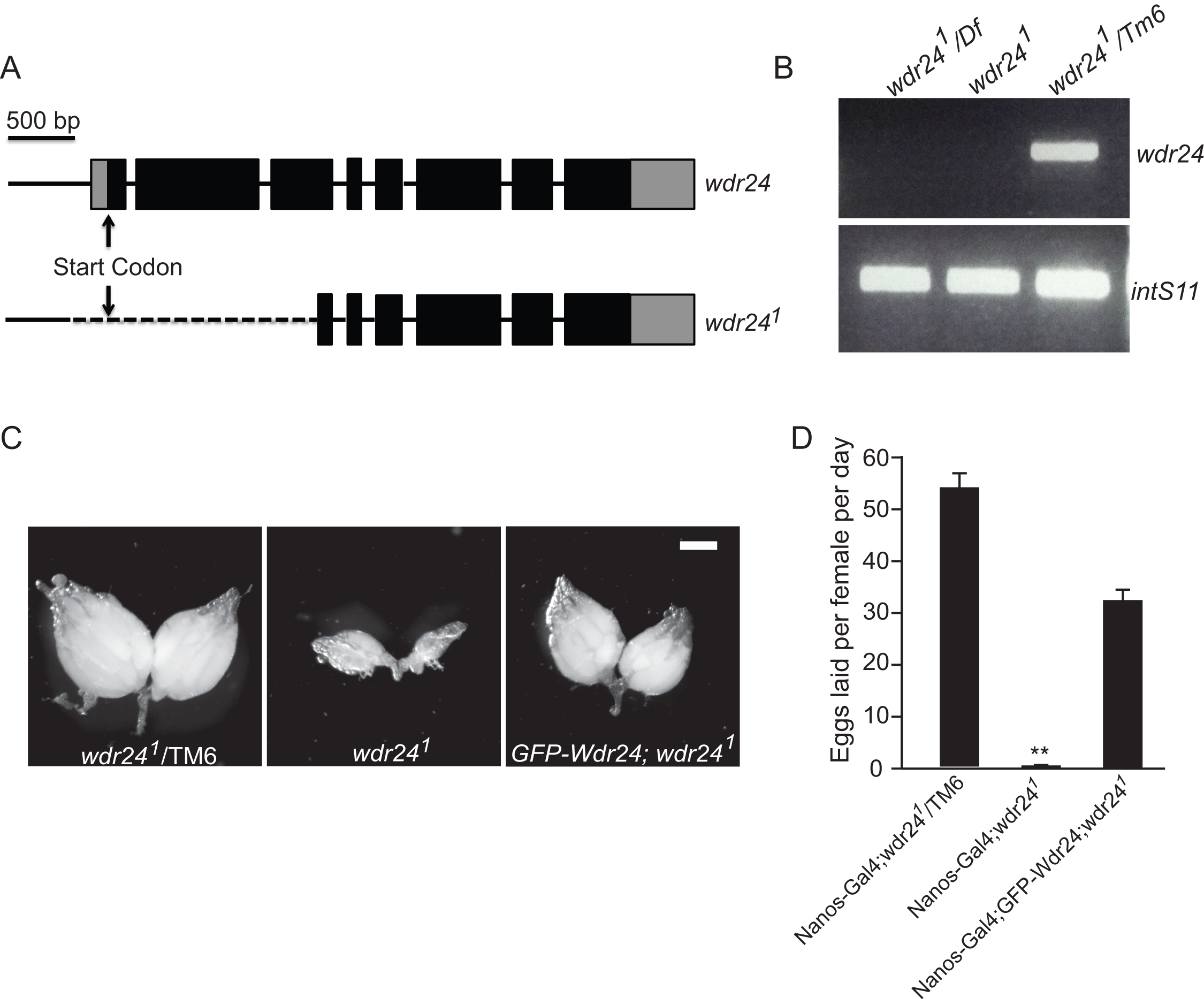

Supplement: S1 Fig — (A) Schematic map shows the wdr241 deletion. Dashed line marks the deletion position. (B) RT-PCR demonstrates that wdr24 mutants do not produce full length mRNA. Integrator subunit 11 (intS11) was used as the internal control in order to demonstrate that the deletion only affects wdr24 gene expression but not the neighboring gene intS11. (C-D) Expression of GFP-Wdr24 using the germline specific Nanos-Gal4 driver rescues the ovarian and fertility phenotypes of wdr24 mutants. (C) Dissected ovaries from control wdr241/TM6, wdr241 and Nanos-Gal4; GFP-Wdr24; wdr241 females. Size bar is 100 μm. (D) Bar graph shows the number of eggs laid by Nanos-Gal4; wdr241/TM6, Nanos-Gal4; wdr241 and Nanos-Gal4; GFP-Wdr24; wdr241 females. Error bars represent the standard deviation for three independent experiments. ** p value < 0.01. (TIF) [file pgen.1006036.s001.tif]

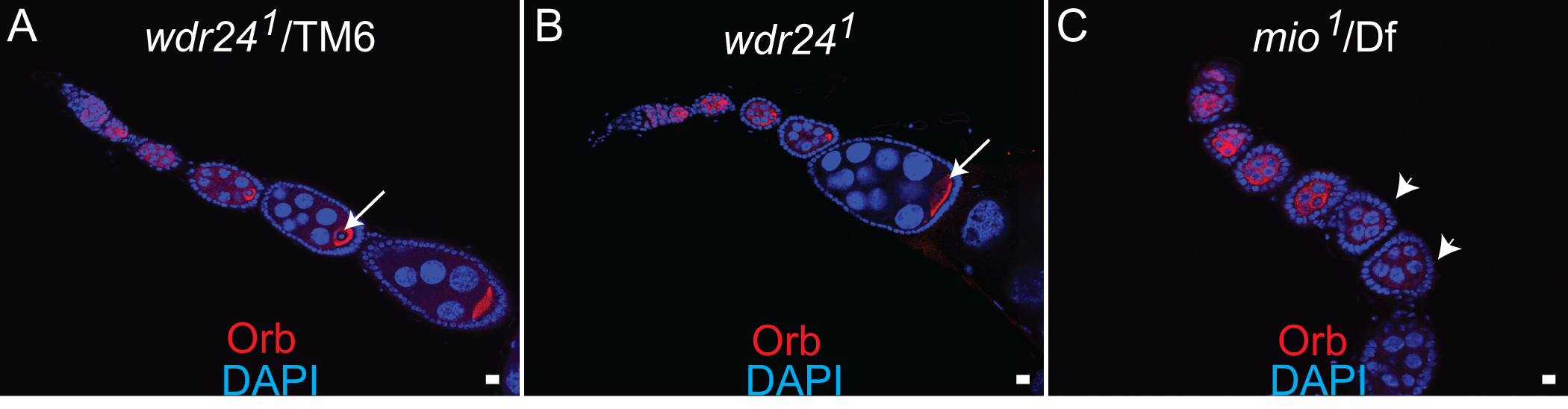

Supplement: S2 Fig — Wdr24 is not required for the maintenance of the oocyte fate. Ovaries stained with DAPI (DNA, blue) and Orb antibody (oocyte marker, red). (A) wdr241/TM6 (B) wdr241 (C) mio1/Df. Note that the wdr241/TM6 and wdr241 mutant egg chambers all contain ooctyes (white arrow), but multiple mio1/Df egg chambers have no apparent oocyte (white arrowhead). Size bar is 10 μm. (TIF) [file pgen.1006036.s002.tif]

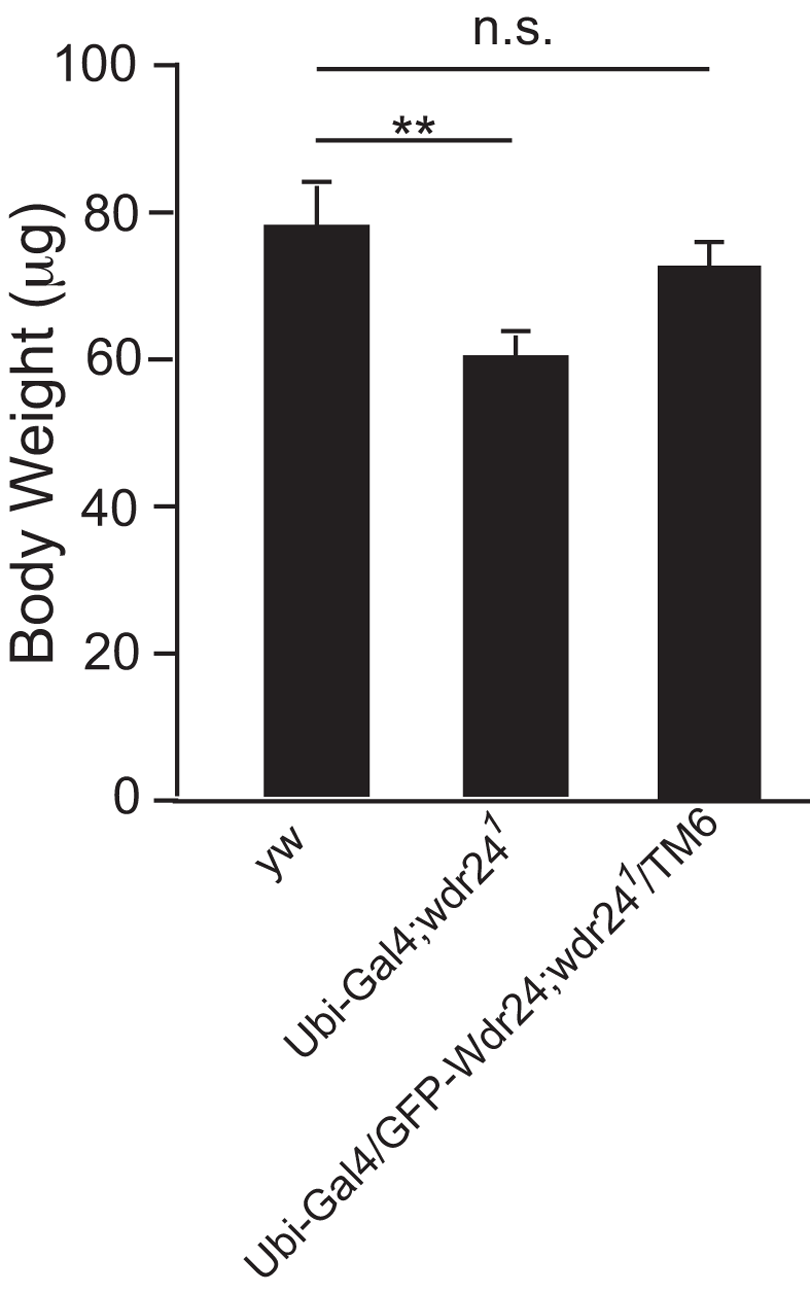

Supplement: S3 Fig — Bar graph shows that overexpression of GFP-Wdr24 using the Ubi-Gal4 driver in wdr241 mutant background significantly increases body weight. ** p value < 0.01. n.s. indicates not significant. (TIF) [file pgen.1006036.s003.tif]

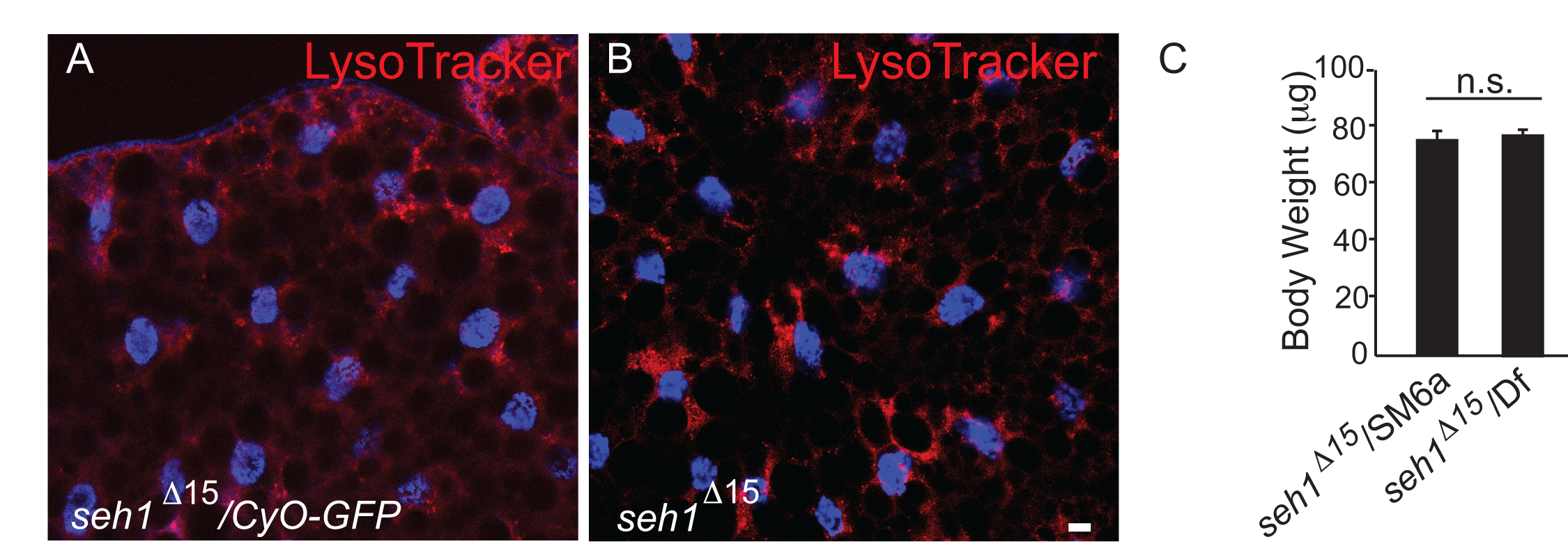

Supplement: S4 Fig — Fat bodies form seh1Δ15/CyO-GFP (A) and seh1Δ15 (B) third instar larvae stained with LysoTracker and Hoechst. Size bar is 10 μm. (C) Quantification of body weights of seh1Δ15/SM6a and seh1Δ15/Df adult males. Error bars represent the standard deviation for three sets of experiments (8 male flies per group). n.s. indicates not significant. (TIF) [file pgen.1006036.s004.tif]

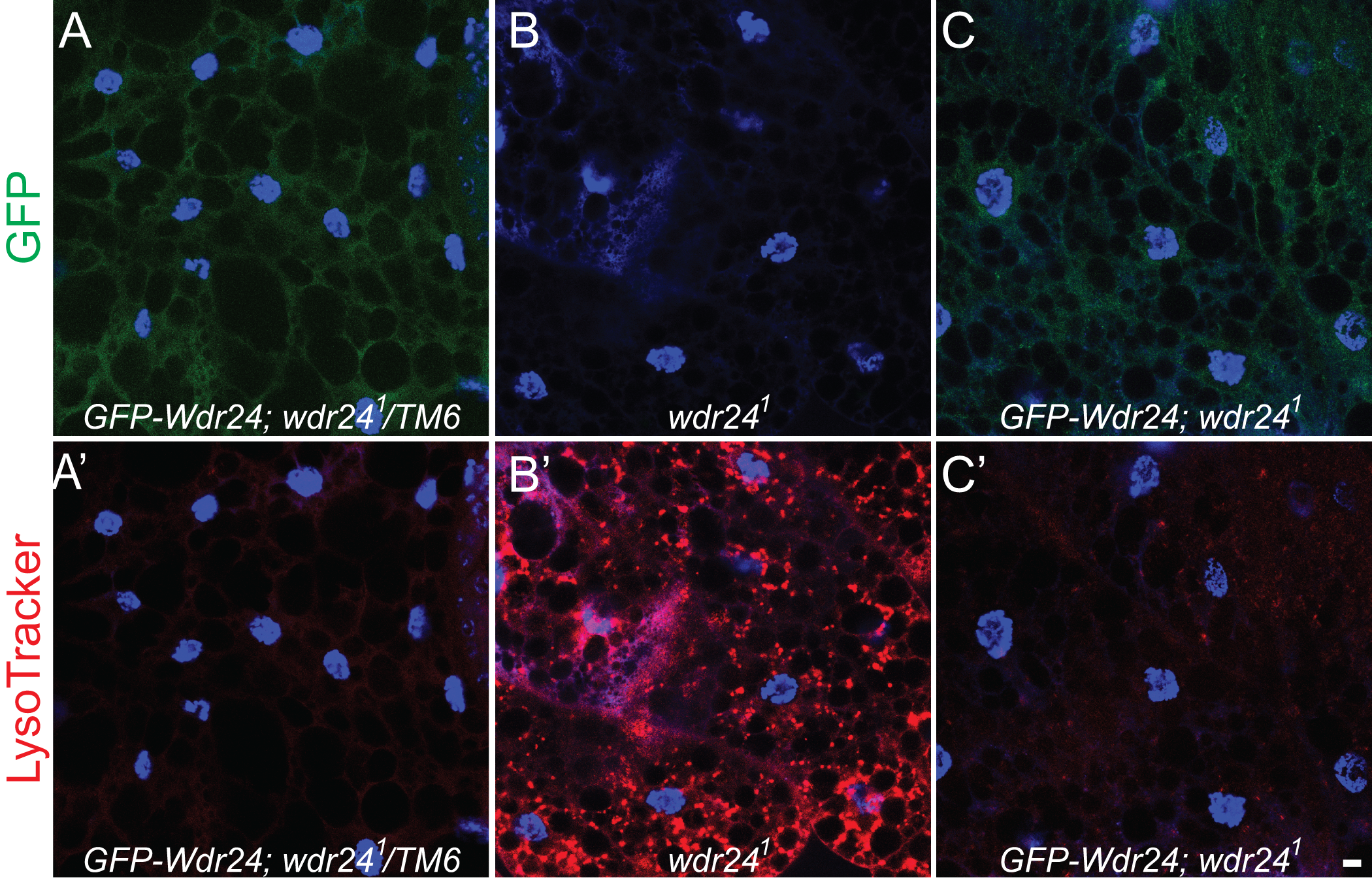

Supplement: S5 Fig — (A-C’) Expression of GFP-Wdr24 using the Cg-Gal4 fat body driver in wdr241 mutant background rescues the LysoTracker accumulation phenotype. Fat bodies from GFP-Wdr24; wdr241 /TM6 (A and A’), wdr241 (B and B’) and GFP-Wdr24; wdr241 (C and C’) third instar larvae were stained with LysoTracker and Hoechst. Size bar is 10 μm. (TIF) [file pgen.1006036.s005.tif]

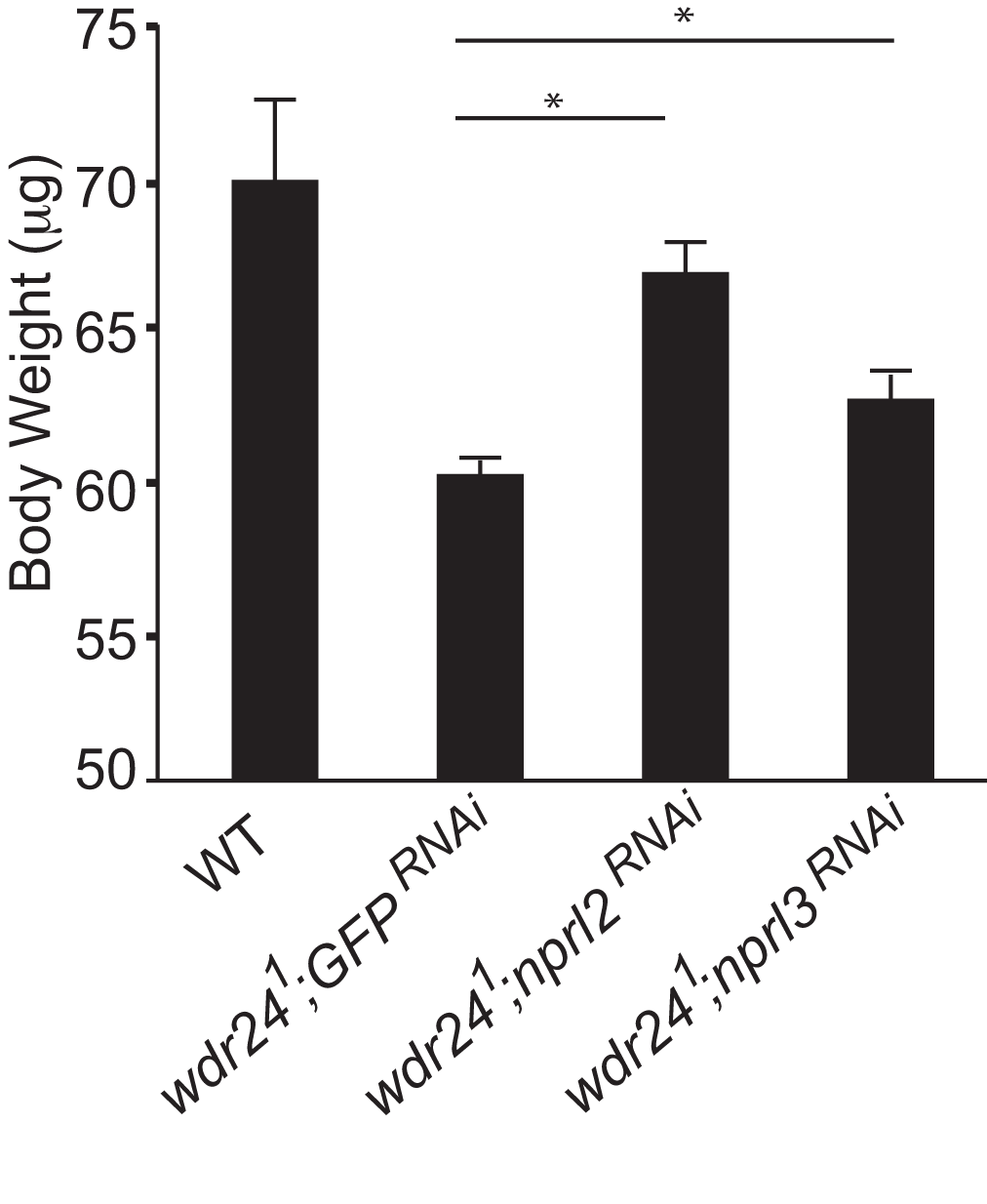

Supplement: S6 Fig — Bar graph shows that depleting nprl2 and nprl3 in the wdr241 mutant background results in an increased body weight. GFPRNAi; wdr241 served as a negative control. * p value < 0.05. (TIF) [file pgen.1006036.s006.tif]

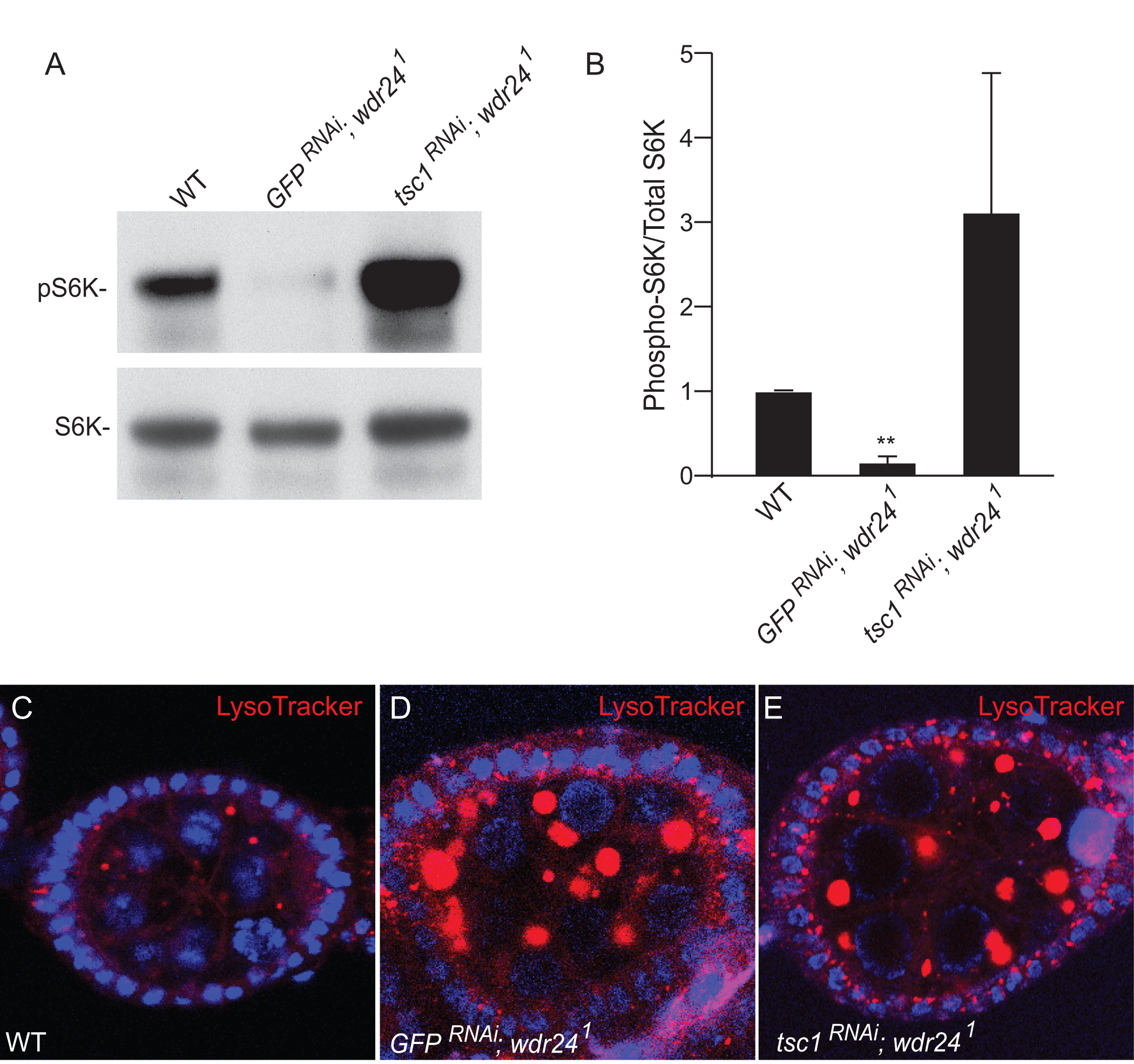

Supplement: S7 Fig — (A) Proteins isolated from WT, GFPRNAi; wdr241 ovaries and tsc1RNAi; wdr241 were analyzed by Western blot probed with pS6K and S6K antibodies. (B) Quantification of phospho-S6K levels relative to the total S6K. Error bars represent the standard deviation for three independent experiments. ** p value < 0.01. (C-E) Depleting tsc1 fails to rescue the lysosomal phenotype in wdr241 ovaries. Ovarioles from WT (C) GFPRNAi; wdr241 (D) and tsc1RNAi; wdr241 (E) females were stained with LysoTracker and Hoechst. Size bar is 10 μm. (TIF) [file pgen.1006036.s007.tif]

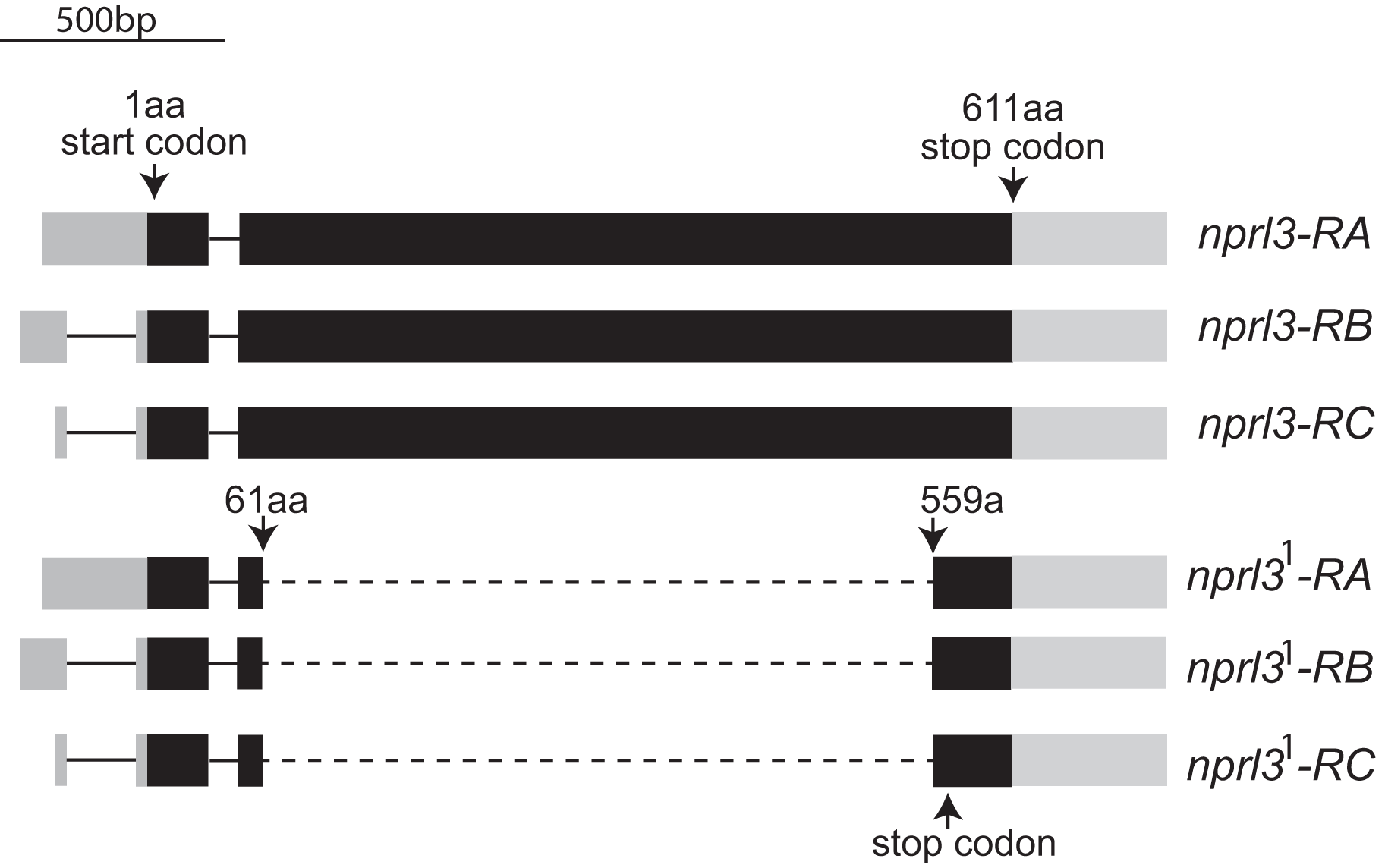

Supplement: S8 Fig — Schematic map shows the deletion end points of the nprl31 allele. Dashed line marks the deletion position. N-terminal break point starts at 61th amino acid from start codon. The deletion causes frame shift and generates a new stop codon after 5 amino acids from the C-terminal break point. (TIF) [file pgen.1006036.s008.tif]

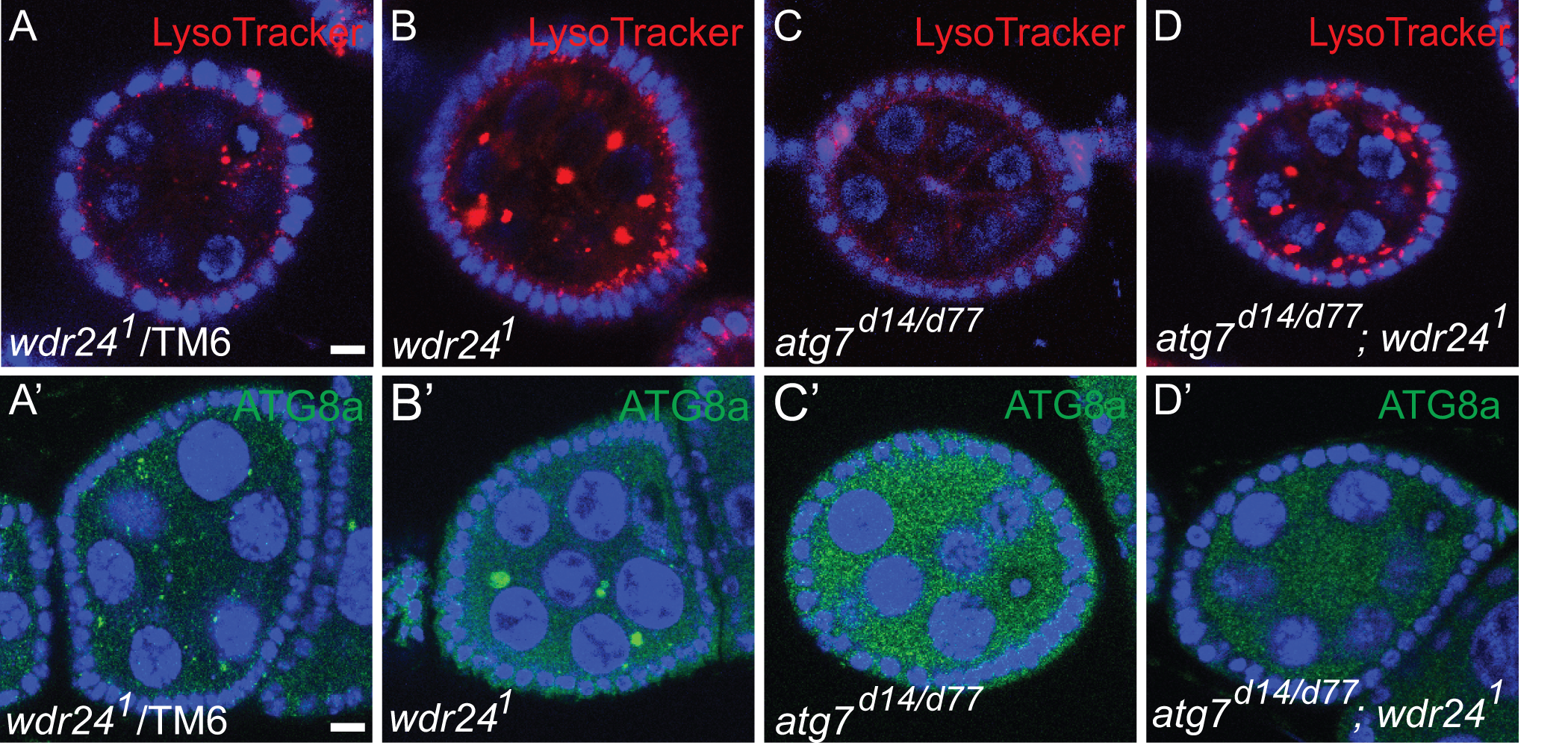

Supplement: S9 Fig — (A-D’) Ovarioles from wdr241/TM6 (A, A’), wdr241 (B, B’), atg7d14/d77 (C, C’) and atg7d14/d77; wdr241 (D, D’) females stained with LysoTracker, anti-Atg8a and Hoechst or DAPI. Note that atg7;wdr24 double-mutant ovaries accumulate lysotracker positive puncta that are not positive for the autophagy marker ATG8a. Size bar is 10 μm. (TIF) [file pgen.1006036.s009.tif]

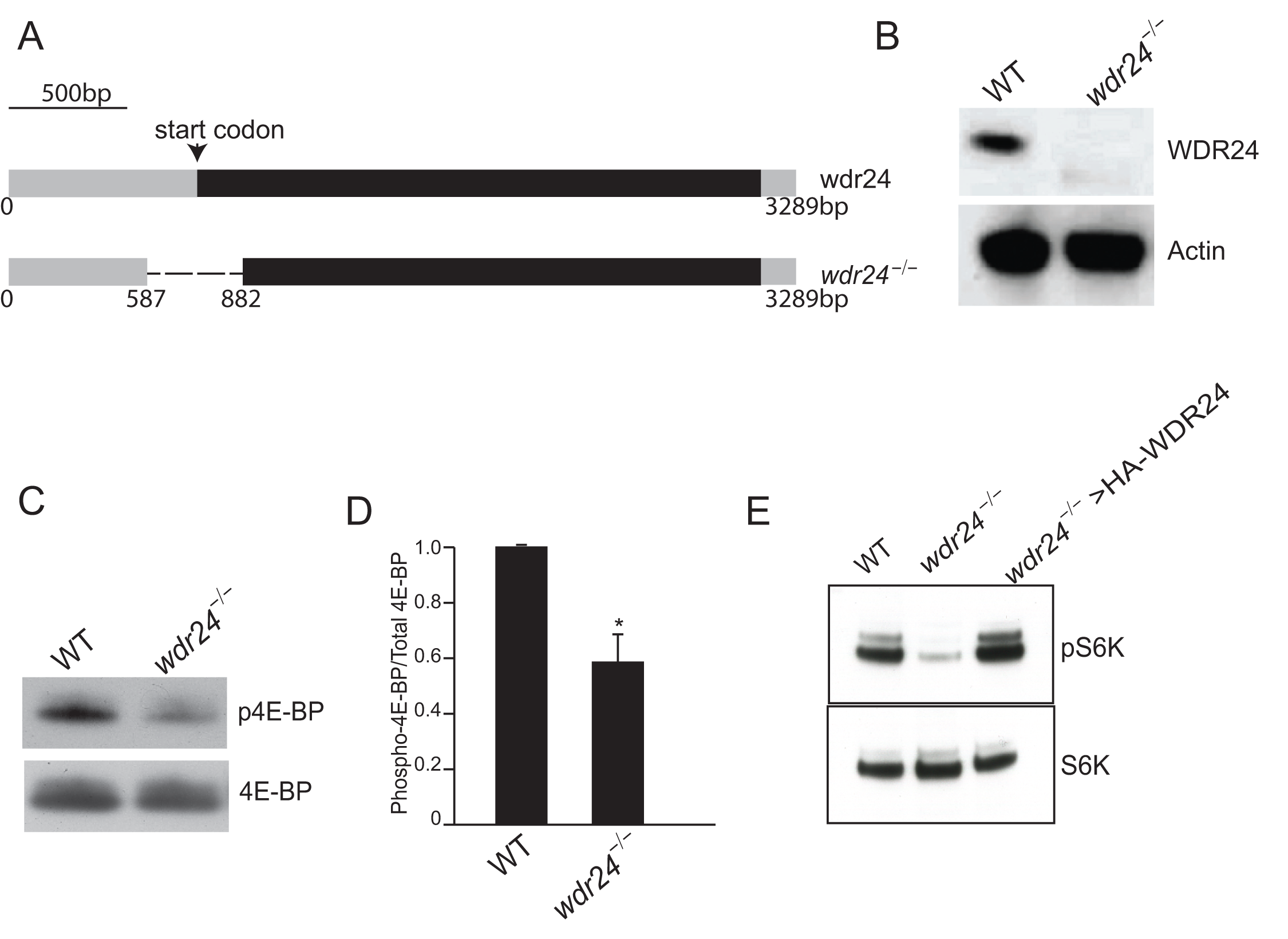

Supplement: S10 Fig — (A) Schematic map shows the position of wdr24-/- deletion. (B) Western blot of wild type (WT) and wdr24-/- probed with WDR24 and actin antibodies. Note that the wdr24-/- mutant cells do not express the WDR24 protein. (C) Western blot of wild type (WT) and wdr24-/- probed with phospho-4E-BP and 4E-BP antibodies. Note that the wdr24-/- mutant cells have lower phosphor-4E-BP level suggesting a decrease of mTORC1 activity. This experiment has been done in triplicates. (D) Quantification of phospho-4E-BP levels relative to the total 4E-BP. Error bars represent the standard deviation for three independent experiments. * p value < 0.05. (E) Western blot of cell lysates from WT, wdr24-/- and wdr24-/- HA-WDR24 rescued cells probed with antibodies against pS6K and S6K. Note that the overexpression HA tagged WDR24 protein in wdr24-/- mutants increases mTORC1 activity as indicated by pS6K levels. (TIF) [file pgen.1006036.s010.tif]

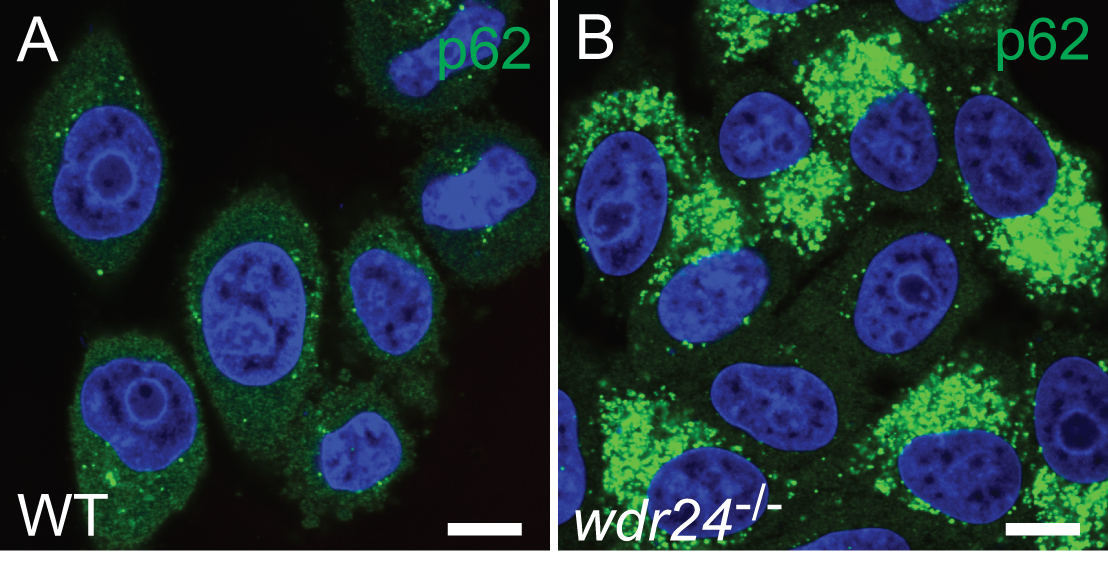

Supplement: S11 Fig — (A and B) Wild type (WT) (A) and wdr24-/- (B) HeLa cells were stained with p62 antibody and DAPI. Size bar is 10 μm. (TIF) [file pgen.1006036.s011.tif]

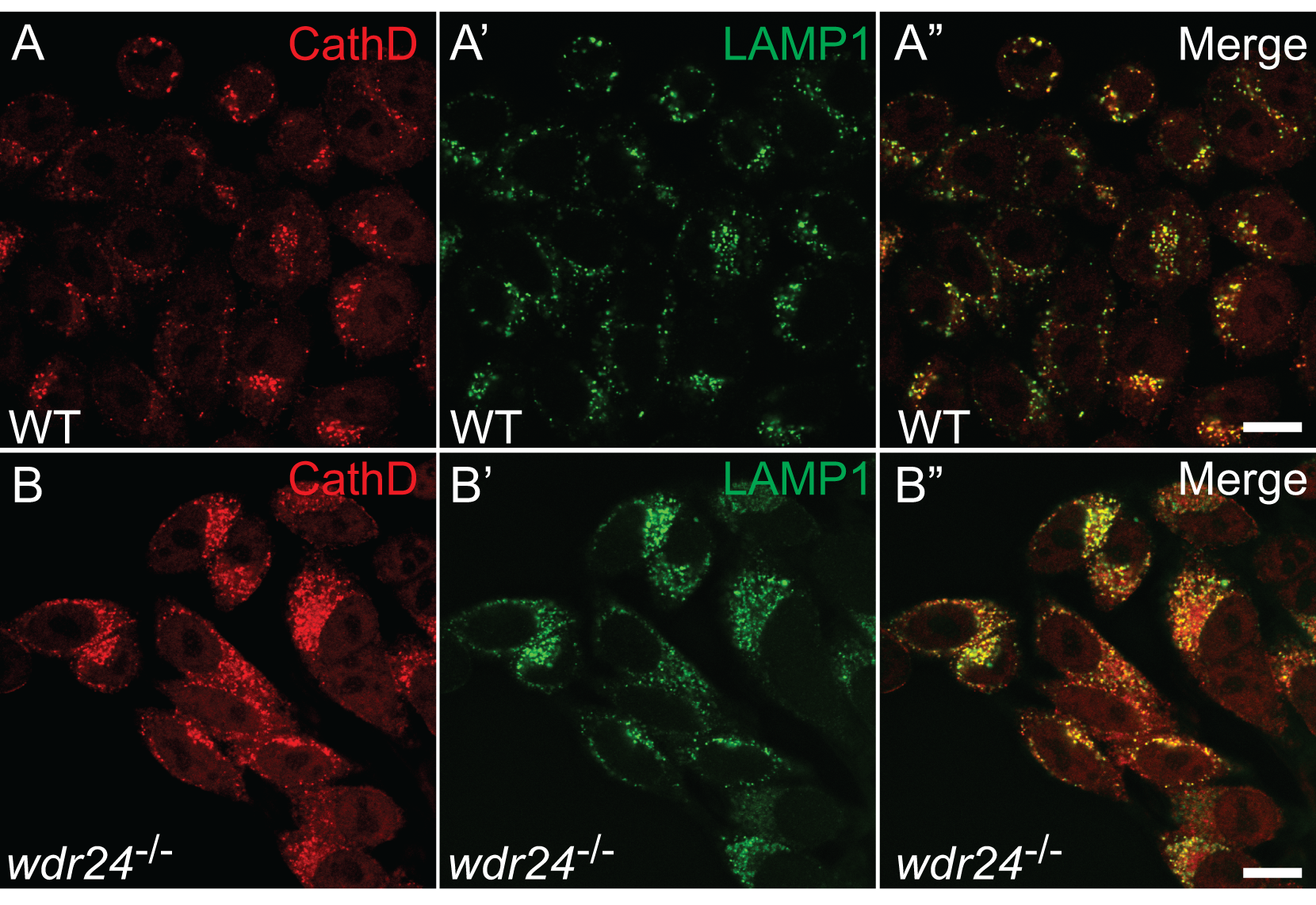

Supplement: S12 Fig — (A–B”) Wild type (WT) (A-A”) and wdr24-/- (B-B”) HeLa cells were stained with Cathepsin D and LAMP1 antibodies. Size bar is 10 μm. (TIF) [file pgen.1006036.s012.tif]

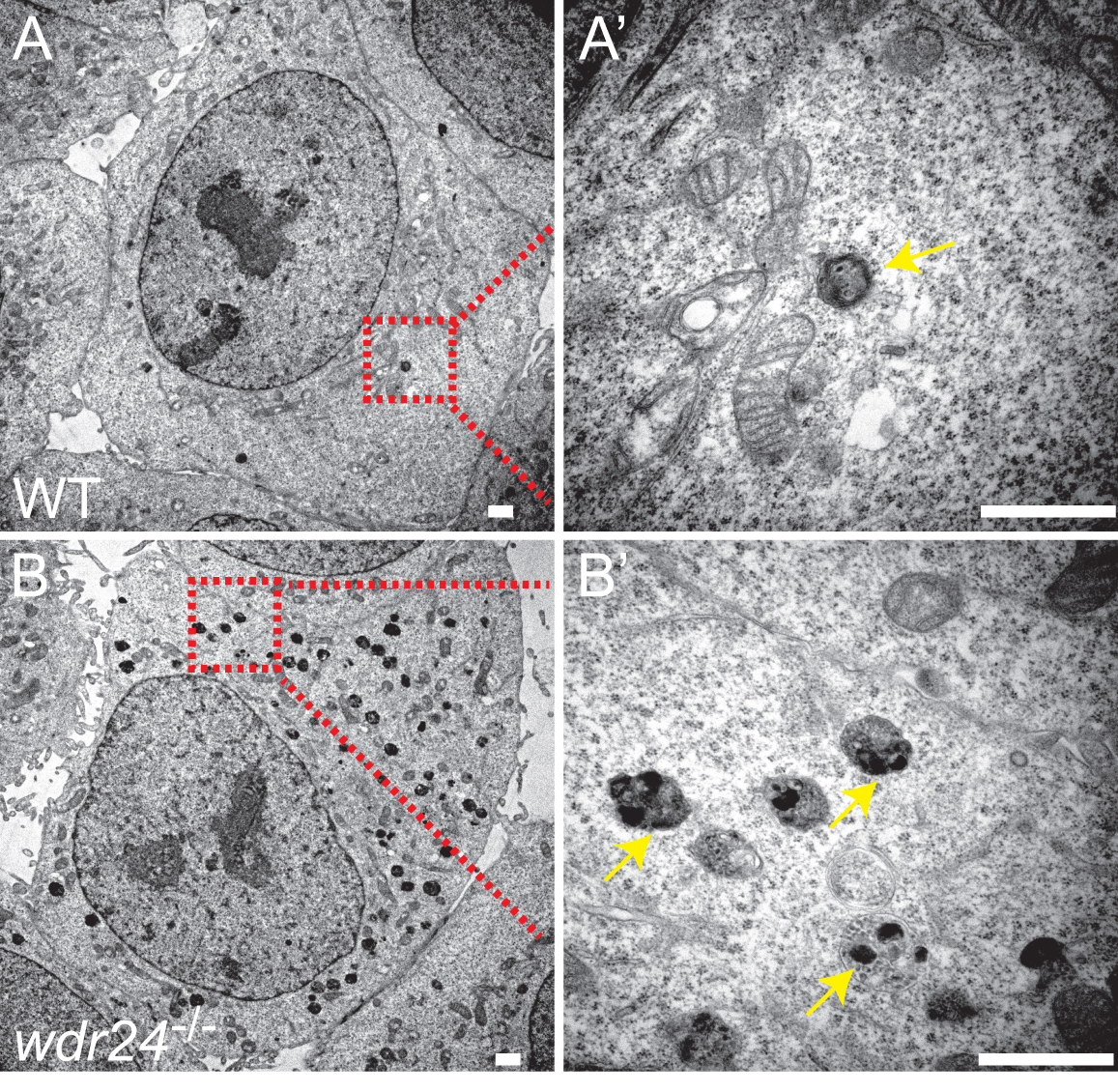

Supplement: S13 Fig — (A-B’) TEM images of lysosomes or autolysosomes from WT (A) and wdr24-/- (B) cells. Autolysosomes are shown at higher magnification for both WT (A′) and wdr24-/- (B′) mutant HeLa cells. Yellow arrows mark autolysosomes. Size bar is 1 μm. (TIF) [file pgen.1006036.s013.tif]
