# Supplementary material for: The GATOR2 Component Wdr24 Regulates TORC1 Activity and Lysosome Function
Source: PLoS Genet. 2016 May 11;12(5):e1006036. doi: 10.1371/journal.pgen.1006036 (PMC4864241; doi:10.1371/journal.pgen.1006036)
Supplement: S1 Table — (DOCX) [file pgen.1006036.s014.docx]

**S1 Table. Identification of the GATOR2 complex in *Drosophila.***

| Protein Name | Isolated Peptides | Unique Peptides |
| --- | --- | --- |
| Mio | 68 | 28 |
| Seh1(Nup44A) | 45 | 20 |
| CG12090(Iml1) | 14 | 13 |
| CG8783(Nplr3) | 11 | 10 |
| CG9104(Nplr2) | 8 | 5 |
| CG7609(Wdr24) | 8 | 7 |
| CG4705(Wdr59) | 8 | 6 |
| Sec13 | 5 | 4 |
