# Supplementary material for: The GATOR2 Component Wdr24 Regulates TORC1 Activity and Lysosome Function
Source: PLoS Genet. 2016 May 11;12(5):e1006036. doi: 10.1371/journal.pgen.1006036 (PMC4864241; doi:10.1371/journal.pgen.1006036)
Supplement: S2 Table — (DOCX) [file pgen.1006036.s015.docx]

| **Primers** | **Sequence** |
| --- | --- |
| *wdr24* CDS Forward | ATGGTACCGAATGCCCGATTCCGTGGAG |
| *wdr24* CDS Reverse | CGCTCGAGTCATGAGTACGCGCAAAGGT |
| *wdr24* CDS Reverse nonstop | CGCTCGAGTAATGAGTACGCGCAAAGGT |
| *wdr24* RT-PCR Forward | ATGGTACCGAATGCCCGATTCCGTGGAG |
| *wdr24* RT-PCR Reverse | CCCTCGAGTCAGGGTTCATACTCCTTGAT |
| *Ints11* RT-PCR Forward | GACGAATTCAACCTGGAAAC |
| *Ints11* RT-PCR Reverse | CTAGCACATATTCTGCAGCA |
| Crispr-*wdr24*-2 Forward | CACCGTATCAGCCAATCAGCCTGAC |
| Crispr-*wdr24*-2 Reverse | AAACGTCAGGCTGATTGGCTGATAC |
| Crispr-*wdr24*-3 Forward | CACCGTACGGCCTGCCACGACCACC |
| Crispr-*wdr24*-3 Reverse | AAACGGTGGTCGTGGCAGGCCGTAC |
| Crispr-Sequence-*wdr24* Forward | CTCGCTCCCCTGCTGGGATTGACT |
| Crispr-Sequence-*wdr24* Reverse | CACATGCCGTCCAGCCCAACTTGC |
| Crispr-*nprl3*-3F | CTTCGCTTGTGAAGCTGTATCAGA |
| Crispr-*nplr3*-3R | AAACTCTGATACAGCTTCACAAGC |
| Crispr-*nprl3*-5F | CTTCGGCTCTTGGCAGCACCCAAG |
| Crispr-*nprl3*-5R | AAACCTTGGGTGCTGCCAAGAGCC |

**S2 Tables. Primer list**
